# Acute and long-term cardioprotective effects of the Traditional Chinese Medicine MLC901 against myocardial ischemia-reperfusion injury in mice

**DOI:** 10.1038/s41598-017-14822-x

**Published:** 2017-10-31

**Authors:** Anne Vincent, Aurélie Covinhes, Christian Barrère, Laura Gallot, Soulit Thoumala, Christophe Piot, Catherine Heurteaux, Michel Lazdunski, Joël Nargeot, Stéphanie Barrère-Lemaire

**Affiliations:** 1 IGF, CNRS, INSERM, Univ Montpellier, Montpellier, France; 2 Laboratory of Excellence Ion Channel Science and Therapeutics, Valbonne, France; 3 Département de cardiologie interventionnelle, Clinique du Millénaire, Montpellier, France; 4 IPMC, UMR CNRS 7275, Univ Côte d’Azur, Valbonne, France

## Abstract

MLC901, a traditional Chinese medicine containing a cocktail of active molecules, both reduces cerebral infarction and improves recovery in patients with ischemic stroke. The aim of this study was to evaluate the acute and long-term benefits of MLC901 in ischemic and reperfused mouse hearts. *Ex vivo*, under physiological conditions, MLC901 did not show any modification in heart rate and contraction amplitude. However, upon an ischemic insult, MLC901 administration during reperfusion, improved coronary flow in perfused hearts. *In vivo*, MLC901 (4 µg/kg) intravenous injection 5 minutes before reperfusion provided a decrease in both infarct size (49.8%) and apoptosis (49.9%) after 1 hour of reperfusion. Akt and ERK1/2 survival pathways were significantly activated in the myocardium of those mice. In the 4-month clinical follow-up upon an additional continuous *per os* administration, MLC901 treatment decreased cardiac injury as revealed by a 45%-decrease in cTnI plasmatic concentrations and an improved cardiac performance assessed by echocardiography. A histological analysis revealed a 64%-decreased residual scar fibrosis and a 44%-increased vascular density in the infarct region. This paper demonstrates that MLC901 treatment was able to provide acute and long-term cardioprotective effects in a murine model of myocardial ischemia-reperfusion injury *in vivo*.

## Introduction

Cardiovascular diseases are still the leading cause of morbidity and mortality worldwide, especially in industrialized countries. Acute myocardial infarction (AMI) is mainly due to the occlusion of a coronary artery inducing an ischemic insult and tissue necrosis. The prognosis of myocardial infarction, complicated by severe arrhythmias and heart failure episodes, is directly related to infarct size. The extent of scar depends mainly on the duration of ischemia. The challenge therefore is the earliest reopening of the culprit artery. Myocardial reperfusion, either by angioplasty or thrombolysis, is so far the only approved treatment able of saving the myocardium subjected to ischemia and to improve the prognosis of patients. However, this abrupt reperfusion induces deleterious secondary effects called ischemia-reperfusion (IR) injury that add to ischemic damage and further increase infarct size^1^_._ The sudden return of blood flow precipitates the death of ischemic myocardial cells by activation of a cascade of complex intracellular events including the release of oxygen free radicals, loss of intracellular and mitochondrial calcium homeostasis, dysfunction of microcirculation, influx of inflammatory cells, edema and apoptotic processes. The damage attributable to IR injury is estimated between 25 and 50% of the total infarct.

Despite an extensive research to target specifically IR injury^2–8^ the only treatment providing efficient cardioprotective effects for AMI patients is early revascularization of the culprit artery. Numerous drugs identified as cardioprotective in animal models have been evaluated in clinical trials and not one drug or product of potential clinical utility has emerged^9,10^. This difficulty of discovering new treatments for patients suffering from myocardial ischemia comes from the failure to translate to humans the strategies developed in animal models. This suggests that other strategies of translation are clearly needed^11^ and that the concept of a “magic bullet” for a “magic single target” is exceeded.

A new hope for targeting myocardial reperfusion injury could emerge from compounds already used in humans as a cocktail of molecules active on different harmful pathways such as traditional Chinese medicine (TCM). Evidence from clinical trials has promoted acceptance of TCM for the treatment of cardiovascular disease^12^. Combination therapy has been used for centuries. Multiple drug-treatment targeting multi-pathways would possess stronger therapeutic efficacies for complex multifactorial diseases^13^.

NeuroAiD™ (MLC601) is a TCM, that is used in China to facilitate recovery after stroke^14^. MLC601 combines 9 plants and 5 animal components. The beneficial effects of MLC601 in cerebral infarction have been well documented both in rodent models and humans^15,16^. The clinical trial CHIMES, which compared MLC601 with placebo in 1099 patients with acute ischemic stroke of intermediate severity, reported a reduction of early recurrent vascular events and vascular deaths in post-stroke patients^14,17,18^. Interestingly, in the Philippine cohort of the CHIMES trial including more patients with predictors of poorer prognosis, functional and neurological outcomes were improved in favor of MLC601^19^. A recent extension study of the CHIMES trial (CHIMES-E) revealed that a 3-month treatment with MLC601 after stroke improves the functional outcome for up to 2 years among patients with cerebral infarction of intermediate severity^20^. A simplified formula called NurAiDII™ (MLC901) composed of only the 9 ingredients of plant origin also displays the same beneficial effects as MLC601 in animal models of focal and global ischemia^21,22^. Clinical assays, including large trials (>1000 patients) on the use of MLC601, have shown that the compound has a good safety profile and a marked efficacy in specific cohorts of patients^23^. Safety for MLC901 was recently demonstrated in a recent pilot study in patients with mild and severe brain trauma injury^24^. Pharmacological data obtained from rodents have demonstrated that, in addition to infarct volume reduction, MLC601 and MLC901 drastically improve recovery of both motor and cognitive functions in focal and global ischemia^16,21^. These beneficial effects have also been observed in an *in vitro* assay of oxygen-glucose deprivation that mimics brain ischemic conditions^25^. Both MLC601 and MLC901 are cocktail of molecules acting on many different targets such as free radical production (inhibition), ATP-sensitive K^+^ channels (activation)^15,16,25^ or HIF1α (Hypoxia-Inducible Factor 1-alpha), EPO (erythropoietin), VEGF (Vascular Endothelial Growth Factor) expression^26^.

Considering (i) that cardiac and brain ischemia share many of the mechanisms that lead to cell death in the two different organs^27^, (ii) that the interesting result of the large clinical study (CHIMES) has shown that MLC601 provides a reduction of early recurrent vascular effects and cardiovascular death in post-stroke patients^28^ and (iii) that MLC901 contains molecules (astragaloside, tanshinone, salvianolic acid, ferulic acid, tetramethylpyrazine) previously shown to be cardio-beneficial^29–33^, we decided to explore the efficacy of MLC901 *in vivo* and *ex vivo* in mice, at the acute phase of myocardial ischemia-reperfusion injury as well as in a long-term study after 4 months following myocardial ischemia in mouse. This paper reports very encouraging beneficial effects of MLC901. The result of such a work is potentially of a great interest since both MLC901 and MLC601 are currently used in patients with stroke, facilitating extension of its use from stroke to cardiac ischemia in humans.

## Results

### Acute effects of MLC901 during myocardial ischemia-reperfusion

Clinical assays have previously shown that both MLC601 and MLC901 are safe in humans^23,24^. We showed that MLC901 administration does not affect the basic cardiac physiological parameters. To do this, heart rate and contraction amplitude were recorded on isolated mouse hearts perfused with MLC901 *ex vivo*. In addition, *in vitro* experiments on isolated mouse ventricular cells were performed in order to measure the changes in sarcomere length as an index of contractile performance (Supplementary Figs S1 and S2).

**Figure 1:**
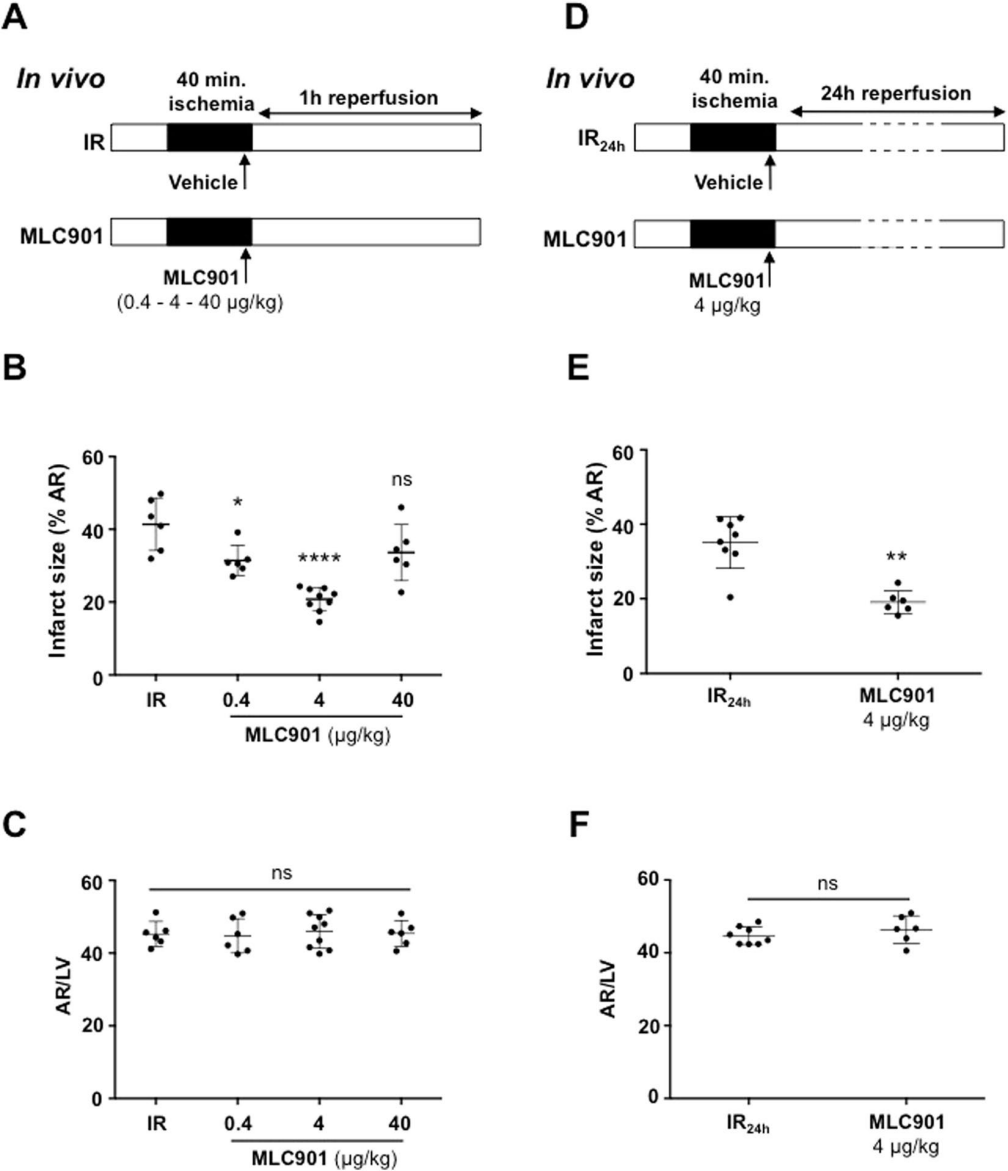
Cardioprotection induced by MLC901 *in vivo* at the acute phase of myocardial ischemia-reperfusion. (**A,D**) Experimental protocols: mice underwent a surgical protocol of myocardial 40 min-ischemia followed by 1h-reperfusion (IR; panel A) or 24h-reperfusion (IR_24h_; panel D). Injection of MLC901 (0.4, 4 or 40 µg/kg) was performed intravenously 5 minutes before the onset of reperfusion. Infarct size measurement was performed at the end of reperfusion. **(B)** Scatter dot blots and means ± SD were plotted for infarct size (in % of area at risk). When mice were treated with MLC901 at 4 µg/kg, a 49.8%-decrease in infarct size was observed. A smaller cardioprotective effect was observed for the treatment with 0.4 µg/kg MLC901. This cardioprotective effect was not observed with 40 µg/kg MLC901. **(C)** Scatter dot blots and means ± SD were plotted for AR/LV mass. (**D**) IR_24h_ animals received MLC901 (4 µg/kg) or physiological saline serum alone (IR). **(E)** Scatter dot blots and means ± SD were plotted for infarct size (in % of area at risk). **(F)** Scatter dot blots and means ± SD were plotted for AR/LV mass. Statistical analysis was performed using ANOVA test with the Tukey’s *post hoc* test for multiple comparisons or Mann-Whitney test for comparisons between 2 groups.

The cardioprotective effects of MLC901 were evaluated *in vivo* using a mouse model of myocardial ischemia-reperfusion injury. Mice (Table 1) were subjected to 40 minutes of ischemia followed by 1 hour of reperfusion. MLC901 (0.4, 4 or 40 µg/kg) was administered intravenously 5 minutes before the onset of reperfusion (Fig. 1A).

**Table 1:** Baseline characteristics of mice at the time of inclusion.

	Mouse number	Sex	Weight (g)	Age (weeks)
SHAM (n = 8)	Mice #6	male	27	8
	Mice #7	male	28	8
	Mice #15	male	27	8
	Mice # 17	male	27	8
	Mice # 43	male	24	8
	Mice # 45	male	27	8
	Mice # 49	male	27	8
	Mice # 52	male	25	8
IR (n = 20)	Mice # 2	male	24	8
	Mice # 10	male	25	8
	Mice # 12	male	26	8
	Mice # 16	male	26	8
	Mice # 18	male	26	8
	Mice # 19	male	26	8
	Mice # 22	male	26	8
	Mice # 24	male	24	8
	Mice # 26	male	26	8
	Mice # 27	male	26	8
	Mice # 28	male	26	8
	Mice # 32	male	25	8
	Mice # 33	male	24	8
	Mice # 34	male	25	8
	Mice # 36	male	25	8
	Mice # 37	male	25	8
	Mice # 38	male	25	8
	Mice # 39	male	24	8
	Mice # 44	male	24	8
	Mice # 51	male	24	8
MLC901 (n = 18)	Mice # 1	male	23	8
	Mice # 3	male	25	8
	Mice # 5	male	26	8
	Mice # 11	male	25	8
	Mice # 14	male	24	8
	Mice # 20	male	27	8
	Mice # 25	male	24	8
	Mice # 29	male	25	8
	Mice # 30	male	25	8
	Mice # 35	male	25	8
	Mice # 40	male	24	8
	Mice # 41	male	25	8
	Mice # 47	male	27	8
	Mice # 50	male	27	8
	Mice # 53	male	27	8
	Mice # 54	male	27	8
	Mice # 55	male	22	8
	Mice # 56	male	25	8

In the MLC901 group, infarct size was significantly decreased by 24% *versus* IR for the 0.4 µg/kg dose (31.44 ± 4.13, n = 6; *p = 0.03) and by 49.8% for the 4 µg/kg dose (20.80 ± 3.21, n = 9 for MLC901 *versus* 41.39 ± 7.21, n = 6 for IR; ****p < 0.0001) (Fig. 1B). There was no cardioprotective effect when MLC901 was injected at 40 µg/kg (33.61 ± 7.72, n = 6; p = 0.11 *versus* IR). When the duration of reperfusion was prolonged to 24 hours (Fig. 1D) a significant 45.5%-decrease (19.13 ± 3.00, n = 6 *versus* 35.11 ± 6.93, n = 8; **p = 0.001) in infarct size was observed when mice were treated at 4 µg/kg, the dose providing the maximal cardioprotective effects (Fig. 1E). No statistical difference among groups was noted for the values of area at risk expressed as a percentage of the left ventricular mass (p = 0.96; Fig. 1C and p = 0.35; Fig. 1F).

### Long-term cardioprotective effects of MLC901

**Figure 2:**
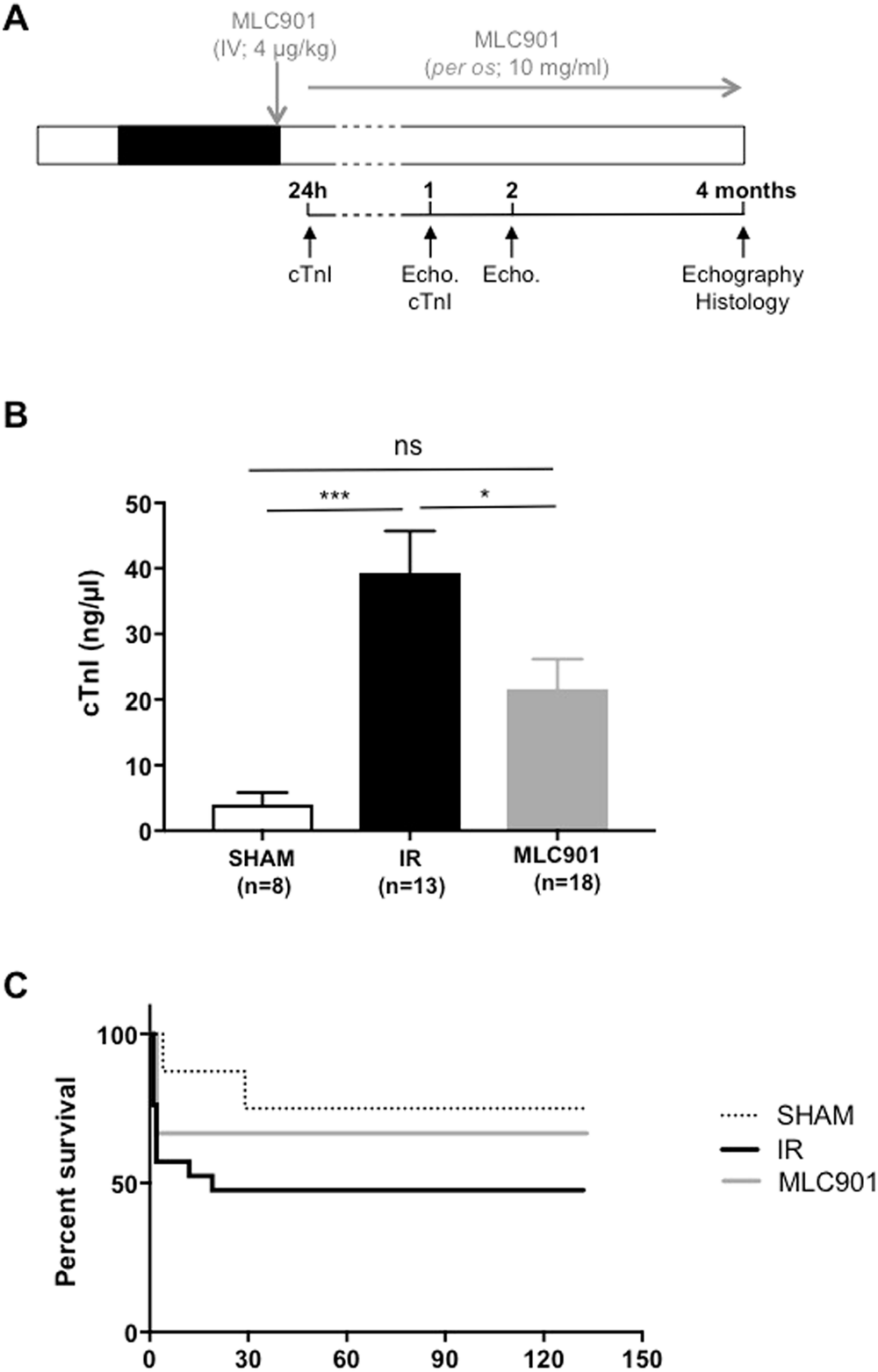
Evaluation of long-term cardioprotective effects. (**A**) Experimental protocol of myocardial ischemia-reperfusion (IR_4m_). MLC901 solution was administered intravenously 5 minutes before the onset of reperfusion (4 µg/kg) and in the drinking water (10 mg/ml) during 4 months. Blood samples were collected at 24 h and 1 month post-surgery. Echocardiographic recordings were performed at 1, 2 and 4 months. At the end of the protocol, a histological analysis was performed. (**B**) Plasmatic fractions were used to quantify cTnI level using the high sensitivity mouse cardiac Troponin-I Elisa. Statistical analysis was performed using ANOVA test with the Tukey’s *post hoc* test for multiple comparisons. (**C**) Kaplan-Meier survival curves for the 4-months period of follow-up (total of 130 days) (Logrank comparison of the curves: p = ns).

In order to evaluate the long-term cardioprotective effects of MLC901, we used a mouse model of ischemia (50 min)-reperfusion followed by a 4-month period of clinical follow-up (Fig. 2A).

Cardiac troponin I (cTnI) and T isoforms are well established biomarkers used in the clinical setting for the diagnosis and prognosis of cardiac injury and they play a key role in the risk stratification of acute coronary syndromes^34,35^ as well as in mouse animal models^36^. A significant increase in cTnI concentration was observed in the IR group *versus* SHAM (4.00 ± 5.25, n = 8 for SHAM *vs* 39.29 ± 23.18, n = 13 for IR; ***p = 0.0006) evaluated at 24h-post-infarction (Fig. 2B). The treatment with MLC901 (4 µg/kg) was able to significantly prevent this increase by 45% (21.59 ± 19.32, n = 18 for MLC901 *vs* 39.29 ± 23.18, n = 13 for IR, *p = 0.04). At 1-month post-infarction, plasmatic cTnI concentrations were close to zero in all groups as expected. In parallel, we observed a decrease in mortality for mice treated with MLC901 during the 4-month period corresponding to a 33.33% total mortality rate for the MLC901 group *versus* 55% for the IR group (Supplementary Fig. S3) despite a lack of significant difference in the survival proportion during the same period (Fig. 2C).

**Figure 3:**
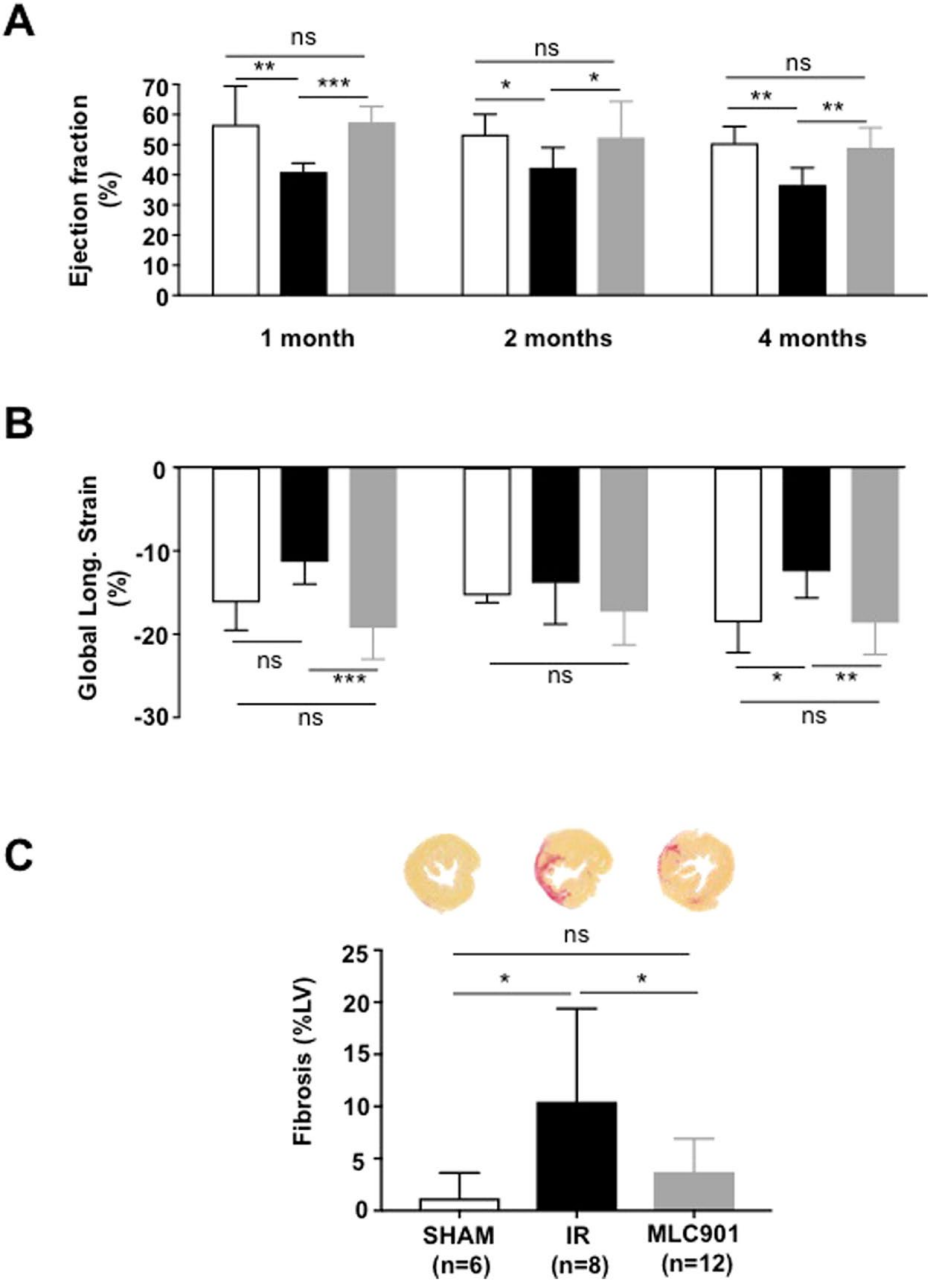
4-month follow-up study of MLC901 treated mice. (**A**) Ejection fraction (%) was assessed by echocardiography from measurements performed on bidimensional images of the parasternal long axis (B-mode) using *Vevolab* software in n = 6 SHAM, n = 7 IR and n = 12 MLC901. (**B)** Evaluation of the global longitudinal strain (peak, %) in parasternal long axis view using *Vevostrain* software (n = 5 SHAM, n = 7 IR and n = 10 MLC901 mice). Statistical significance was tested using Two-way ANOVA (Tukey’s *post hoc* test). (**C**) Fibrosis area (presented as histogram of means ± SD) was 64%-decreased in MLC901-treated *versus* IR LV samples (*p = 0.03). Statistical analysis was performed using ANOVA with the Tukey’s *post hoc* test for multiple comparisons.

Improved cardiac performance is associated to decreased cardiac injury^37^. For this reason, LV systolic function was evaluated using non-invasive echocardiography in mice that survive during the post-infarction period (Supplementary Table S1). Ejection fraction (EF, %), the main parameter reflecting systolic function, was improved by MLC901 *versus* IR during the whole period of follow-up (****p_*treatment*_ < 0.0001; *p_*time*_ = 0.02; p_*interaction*_ ns = 0.75) (Fig. 3A). These results were confirmed by the measurement of shortening fraction and stroke volume, which is an important determinant of cardiac output (Supplementary Fig. S4A,B).

Speckle Tracking Imaging (STI) permits the identification of beneficial effects following rescue therapy on myocardial function before changes in global LV function^38^. In particular, global longitudinal strain, which represents the myocardial deformation from the base to apex, detects the percent change in length of LV and was reported in mice to be closely correlated with infarct size^39^. MLC901 treatment was able to fully prevent the IR-induced decrease in the global longitudinal strain (***p_*treatment*_ = 0.006; p_*time*_ = 0.50; p_*interaction*_ ns = 0.22; Fig. 3B).

Literature describes that infarct size is correlated with cardiac remodelling, which strongly impairs cardiac function after myocardial infarction^40^. Cardiac fibrosis is a critical problem of post-MI ventricular remodeling^41^. In the group of mice subjected to IR, the percentage of fibrosis assessed by Picrosirius staining was 8-fold higher than in the SHAM group (*p = 0.01) (Fig. 3C). In the group of mice treated with MLC901 the percentage of fibrosis in LV samples was 64%-decreased *versus* IR (3.73 ± 3.17, n = 12 for MLC901 *versus* 10.46 ± 8.93, n = 8 for IR; *p = 0.03). No significant difference was observed between the SHAM and the MLC901 groups (3.73 ± 3.17, n = 12 for MLC901 *versus* 1.20 ± 2.45, n = 6 for SHAM; *p* = 0.63; ns).

### Mechanisms of MLC901 induced cardioprotection

**Figure 4:**
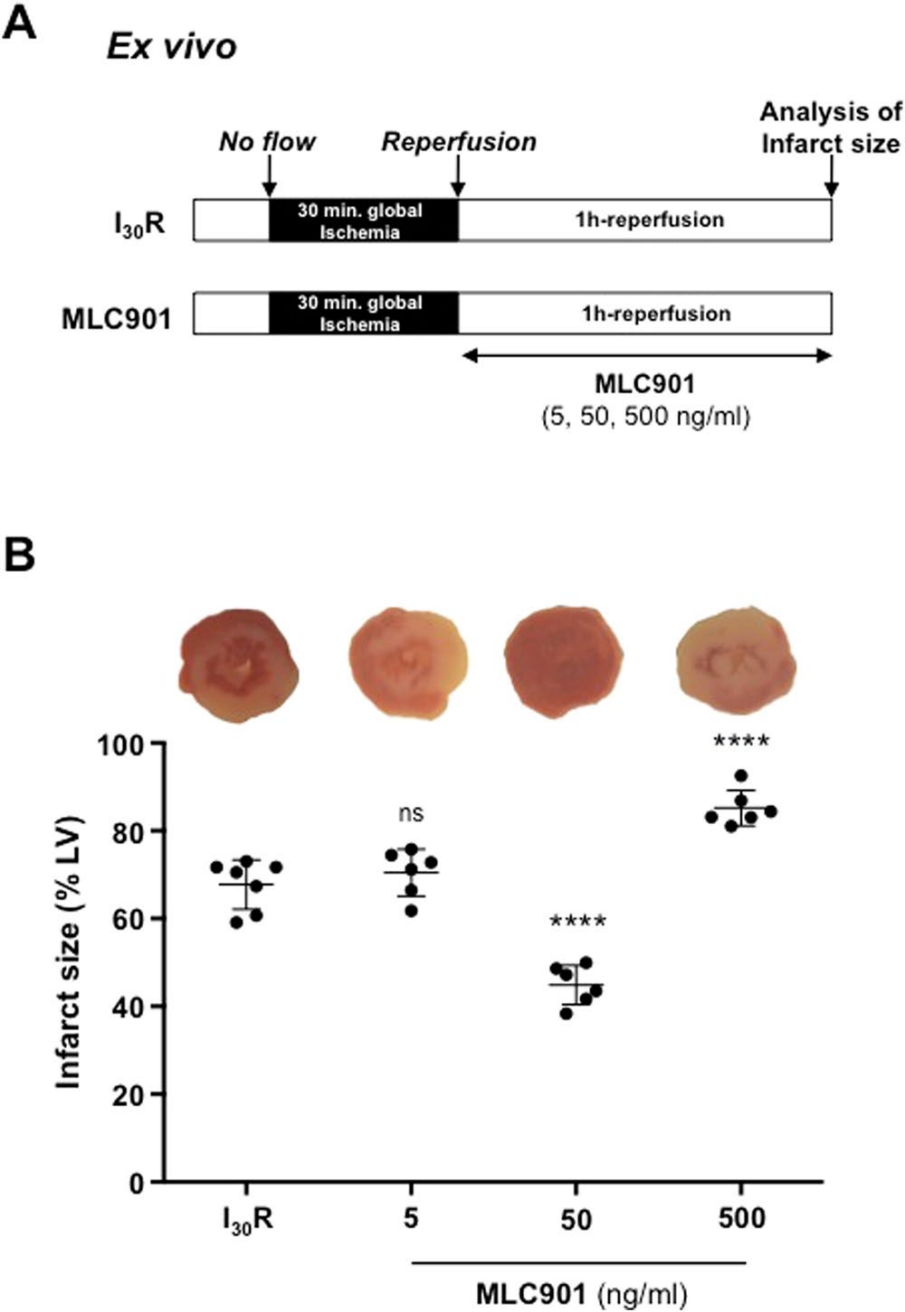
Cardioprotective effect of MLC901 on isolated hearts. **(A)** C57Bl6 mouse hearts were mounted on a Langendorff system. After a 20 minutes period of stabilization, global ischemia was induced during 30 minutes. Reperfusion was achieved by restoring the flow during 60 minutes with the Krebs solution. MLC901 was administered during reperfusion at 3 different concentrations: 5, 50 and 500 ng/ml. (**B**) Scatter dot blots (means ± SD) were represented for infarct size (in % of LV) in IR (n = 7), MLC901 (5 ng/ml, n = 6), MLC901 (50 ng/ml, n = 6), MLC901 (500 ng/ml; n = 6). Representative pictures of TTC-stained LV slices were shown for each group. Statistical analysis was performed using ANOVA with the Tukey’s post hoc test for multiple comparisons.

The protective effects of MLC901 were evaluated on isolated perfused hearts to completely eliminate the neurogenic component. Mouse hearts were subjected to global ischemia (30 minutes) followed by reperfusion (60 minutes) on a Langendorff system perfused with a Krebs solution (I_30_R control, n = 7_;_ Fig. 4A). Treated hearts were subjected to the same ischemic insult and reperfused with MLC901 solution at 5, 50, 500 ng/ml equivalent to the *in vivo* doses (0.4, 4 or 40 µg/kg, respectively). A significant 33.8%-decrease (44.89 ± 4.47, n = 6 for MLC901 *vs* 67.76 ± 5.65, n = 7 for I_30_R; ****p < 0.0001) in infarct size *versus* I_30_R was observed in the group of hearts reperfused with the 50 ng/ml MLC901 solution showing that the cardioprotective is really related to a cardiac mechanism. MLC901 perfused at 5 ng/ml did not induce a cardioprotective effect compared to I_30_R (70.43 ± 5.32, n = 6 for MLC901 *vs* 67.76 ± 5.65, n = 7 for I_30_R; p = 0.77). The highest dose of MLC901 (500 ng/ml) induced an increase in infarct size compared to I_30_R control (85.17 ± 4.07, n = 6 for MLC901 *vs* 67.76 ± 5.65, n = 7 for I_30_R; ****p < 0.0001; Fig. 4B).

**Figure 5:**
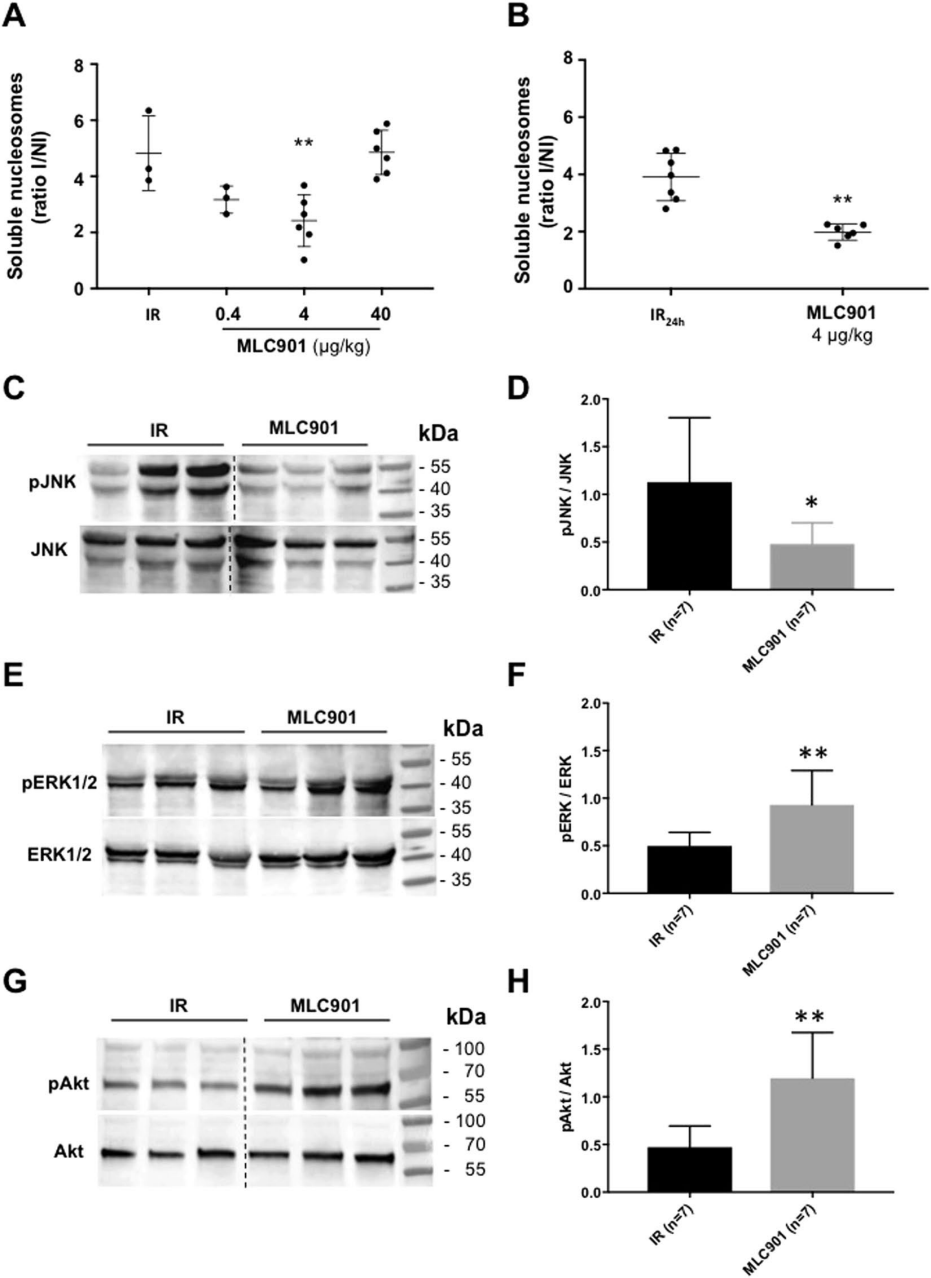
Cardioprotection is mediated by an inhibition of apoptosis *in vivo*. **(A)** Specific DNA fragmentation was quantified after 1h-reperfusion. Injection of MLC901 (0.4, 4 and 40 µg/kg) was performed intravenously 5 minutes before the onset of reperfusion. Scatter dot blots and means ± SD were plotted for the ratio of internucleosomal DNA fragmentation measured in I *versus* NI regions of LV from IR or MLC901-treated mice. **(B)** Scatter dot blots and means ± SD were plotted for the ratio of internucleosomal DNA fragmentation measured in I *versus* NI regions of LV after 24h-reperfusion in mice not treated (IR_24h_) or treated by MLC901 (4 µg/kg). **(C**–**H)** Western blot analysis of pJNK, JNK, pERK1/2, ERK1/2, pAkt and Akt was performed on LV from MLC901 treated *versus* non-treated IR mice. (**C,E,G**) Representative gel blots for LV protein extract were cropped from initial blots presented in Supplementary Figures S5, S6 and S7. Histograms (means ± SD) were plotted for (**D**) pJNK/JNK, (**F)** pERK1/2/ERK1/2 and (**H**) pAKT/AKT ratios for MLC901 (n = 7) *versus* IR (n = 7); Statistical analysis was performed using ANOVA with the Tukey’s *post hoc* test for multiple comparisons or using the Mann-Whitney test for comparison between two groups.

Apoptosis contributes to ischemia-reperfusion injury in the cardiac tissue and is responsible for the loss of cardiomyocytes after reperfusion during acute myocardial infarction. Internucleosomal DNA fragmentation, a hallmark of apoptosis determined by enzyme-linked immunosorbent assay quantification of soluble oligonucleosomes, was evaluated in the ischemic area of LV subjected to ischemia-reperfusion *in vivo*. MLC901 at 4 µg/kg was able to decrease significantly (by 49.9%) specific DNA fragmentation at 1-hour reperfusion (2.42 ± 0.93, n = 6 for MLC901 *vs* 4.83 ± 1.33, n = 3 for IR; **p = 0.01; Fig. 5A). When the duration of reperfusion was prolonged to 24 hours, a 49.2%-decrease (1.99 ± 0.28, n = 6 for MLC901 4 µg/kg *versus* 3.91 ± 0.83, n = 7 for IR_24h_; **p = 0.001) in DNA fragmentation was observed when mice were treated at 4 µg/kg, the dose providing the maximal cardioprotective effects both *in vivo* and *ex vivo* (Fig. 5B).

Ischemia-reperfusion injury as a cellular stress is characterized by the activation of JNK cell death MAPK. The pJNK/JNK ratio was quantified by Western blotting analysis in the LV of mice with or without MLC901 during myocardial IR *in vivo*. MLC901 was able to significantly decrease the pJNK/JNK ratio observed in the IR group (*p = 0.02; Fig. 5C,D).

Cardioprotection is associated with the activation of pro-survival MAPKs. Ratios of pERK1/2/ERK1/2 and pAkt/AKT were evaluated in the LV of mice subjected to IR with or without MLC901 treatment. Phosphorylation of both ERK1/2 and AKT was significantly increased in MLC901-treated LV compared to IR (**p = 0.005 and **p = 0.002, respectively; Fig. 5E–H).

**Figure 6:**
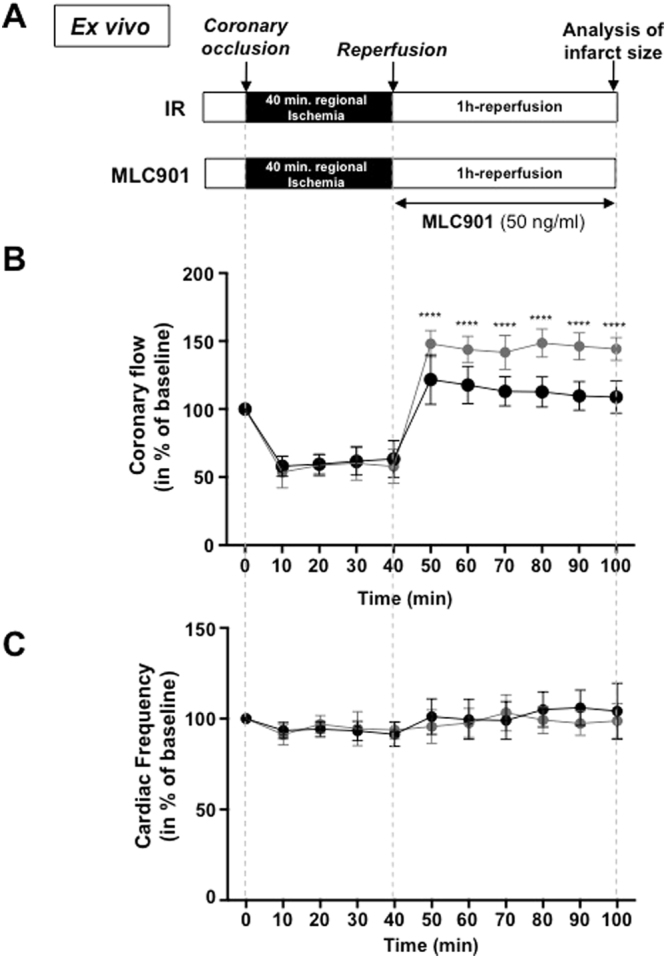
Improved coronary flow in MLC901 treated hearts subjected to IR *ex vivo*. **(A)** Mouse hearts were mounted on an Emka Langendorff system. After a 15 minutes period of stabilization, 40 min. of regional ischemia was applied followed by 1 hour-reperfusion with oxygenated Krebs for control IR hearts (n = 7) or with MLC901 solution at the optimal dose (50 ng/ml) for the MLC901 group (n = 7). All along the protocol, coronary flow and ECG were monitored. Infarct size measurement was performed at the end of reperfusion. **(B,C)** Maximum coronary flow and cardiac frequency were measured each 10 minutes during ischemia and reperfusion. Statistical analysis was performed using two-way ANOVA for repeated measures with a Sidak’s multiple comparisons test. (**D**) Scatter dot blots and means ± SD were plotted for infarct size/AR (in % of area at risk) and AR/LV mass. A 39.8% decrease in infarct size was observed when MLC901 was perfused after ischemia (***p = 0.0006). Statistical analysis was performed using ANOVA test with the Tukey’s *post hoc* test for multiple comparisons or Mann-Whitney test for comparisons between 2 groups.

Myocardial ischemia/reperfusion injury affects not only the cardiomyocyte compartment but also all other cellular compartments, and the coronary circulation has a central role in it^42,43^. In order to evaluate the effect of MLC901 on the coronary flow during ischemia-reperfusion, *ex vivo* experiments were conducted on isolated hearts subjected to regional ischemia followed by reperfusion (Fig. 6A). As shown in Fig. 6B, the coronary flow similarly dropped in a similar way during the ischemic phase (~50%) in both groups. However, a significant increase was observed in MLC901 treated *versus* IR hearts (**p = 0.006). This increase in coronary flow was not accompanied with changes in cardiac frequency (Fig. 6C) while a 39.8%-decrease in infarct size *versus* IR was observed (28.69 ± 4.39, n = 7 for MLC901 *vs* 47.63 ± 3.73, n = 7 for IR, ***p = 0.0006; Fig. 6D). There was no change in the AR/LV ratio among groups (p^ns^ = 0.09; Fig. 6D).

**Figure 7:**
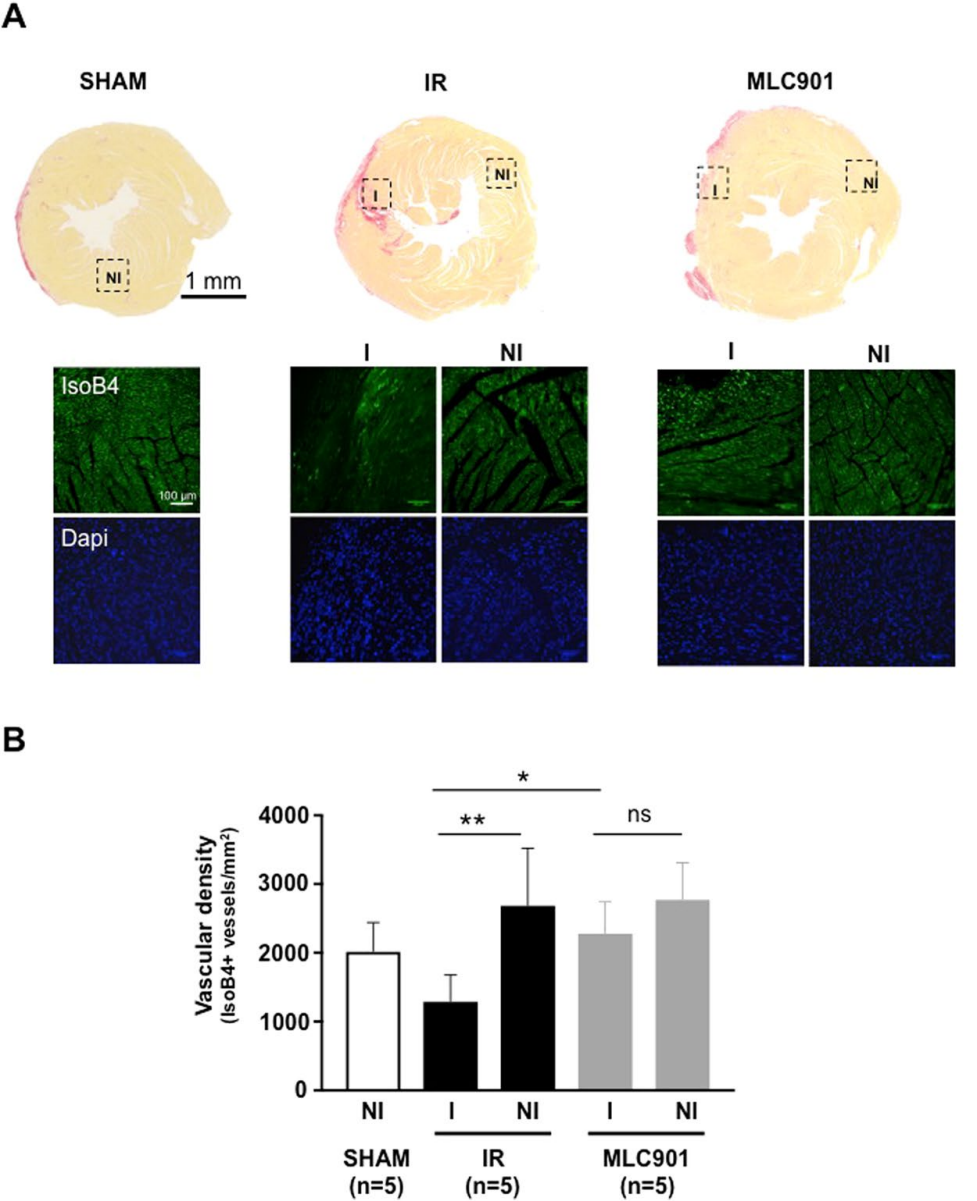
Increased vascular density in the ischemic area in 4 month-MLC901 treated hearts. Immunochemistry was performed on LV sections after 4 months of MLC901-treatment using co-immunostaining with anti-Isolectin B4 and DAPI in order to determine microvascular density in LV tissue. (**A**) Representative pictures of microscopic observations for SHAM, IR and MLC901-treated LV picrosirirus stained section and corresponding enlarged immunostaining images (Original magnification: ×40 oil immersion) showing vessels (isolectinB4, upper panel) and cell nuclei (DAPI, lower panel) taken in the ischemic (I) or non-ischemic region (NI). (**B**) IsolectinB4-positive vessels were determined by randomly counting n = 3 in the ischemic and n = 3 in the non-ischemic areas of both SHAM (n = 5), IR (n = 5) and MLC901 (n = 5).

Since it was previously observed that MLC901 treatment was associated with a pro-angiogenic protective effect after stroke^26^, we decided to quantify the vascular density by immunochemistry, in all hearts, at the end of the long term study. Isolectin B4 positive vessels were counted in the border zone of the infarct and in the non-ischemic remote areas in the LV of all groups of mice. As illustrated in Fig. 7A, there was a significant decrease in the capillary density in the ischemic zone, as compared with the remote non-ischemic region of the LV (I *vs* NI, **p in the IR group). The situation was very different for MLC901-treated mice. In that case, the vascular density was similar in the ischemic zone and in the non-ischemic region (*p = 0.05; Fig. 7B). The MLC901 treatment provided a full compensation of the rarefaction of the vessels observed under IR stress (I *vs* NI, p^ns^ = 0.6 in MLC901 hearts).

## Discussion

This work demonstrates for the first time acute and long-term cardioprotective effects of MLC901 during acute myocardial infarction in mice. First, using a mouse model of myocardial ischemia-reperfusion, our results show that a single dose of MLC901 (4 µg/kg) administered at the onset of reperfusion reduced infarct size by 49.8% *in vivo*, which was correlated with decreased apoptosis in left ventricular tissues. Second, based on a 4-month follow-up study in mice, we have shown that MLC901 provides long-term cardioprotective effects characterized by a preserved cardiac function and a decreased extent in fibrosis. This cardioprotection was confirmed in *ex vivo* experiments indicating that it was mediated by non-neurogenic pathways. Cardioprotection was shown to be related to an anti-apoptotic effect, an improvement of the coronary flow at reperfusion and to a full preservation of the vessel density in the infarct area at 4 months.

Short-term cardioprotective effects (49.8% decrease in infarct size) were shown to occur at the optimal dose of 4 µg/kg when MLC901 was administered intravenously as a single bolus at the onset of reperfusion. The reduction in infarct size was also seen in isolated perfused hearts *ex vivo* suggesting that the mechanisms underlying cardioprotection originate in the cardiovascular tissue.

During myocardial reperfusion, the ischemic myocardium is subjected to several abrupt biochemical and metabolic changes. A plethora of studies have shown apoptotic cell death in different models of animal and human ischemic heart disease^44,45^. In this study, we show that infarct size, specific DNA fragmentation and phosphorylation of JNK1/2 are decreased in MLC901 treated animals after ischemia-reperfusion. These results are to be put in parallel with those previously obtained for brain ischemia. MLC901 is known to also drastically decrease infarct size as well as apoptosis (e.g. DNA fragmentation) in the brain of mice subjected to focal or global ischemia^16,21^.

The PI_3_kinase/Akt and ERK1/2 are part of the RISK (*Reperfusion Injury Salvage Kinase*) pathway which plays a crucial role in preventing reperfusion injury in the myocardium^46^. We examined the expression of protein kinases involved in the RISK pathway including ERK1/2 and PI3kinase-Akt. ERK1/2 activation is associated with protection against apoptosis in cardiac myocytes and from ischemia-reperfusion injury in the heart *in vivo* ^5,46,47^. ERK1/2 phosphorylation was increased in LV tissues from MLC901-treated ischemic mice indicating that TCM compounds protect against ischemic/reperfusion-induced by activating both anti-apoptotic and pro-survival mechanisms as already observed in brain in models of stroke, global ischemia and traumatic brain injury^16,21,48^.

In our 4-month follow-up study, the cTnI level was assessed in all mice as early as 24 h post-MI. A 45%-decrease was observed in mice treated by MLC901 compared to non-treated IR. cTnI is a biomarker for the diagnosis and prognosis of cardiac injury in humans^34,35^. The cTnI plasma level at 24 h was recently identified as an early marker of infarct size in mouse models with permanent ligation of the left ascendant coronary artery^49^ and for ischemia-reperfusion injury^50^. Our results showing a decrease in cTnI plasma level at 24 h in MLC901-treated mice clearly indicate that cardiac injury is decreased at this time point. These results are corroborated by results obtained in our murine model of acute myocardial ischemia-reperfusion, where a 45.5%-decrease in infarct size in the MLC901 treated group was measured in the left ventricle at 24 h of reperfusion.

The infarct size is strongly correlated with cardiac remodelling, which, in turn, impairs cardiac function after myocardial infarction^37,40^. Cardiac fibrosis is a critical character of post-MI ventricular remodeling^41^. Our results show a drastic decrease in fibrosis in the left ventricle of mice treated by MLC901 *versus* the non-treated IR group when evaluated 4 months after cardiac injury. This remodeling involves pathological changes that include chamber dilation, which ultimately progresses to heart failure. Echocardiographical evaluation of the end-systolic and end-diastolic volumes during the 4-month period of follow-up revealed LV dilatation in the IR group. Increased end-systolic and end-diastolic volumes are correlated with a bad prognosis and more specifically, LV end-systolic volume, which is a strong predictor of adverse outcomes^51^. In our study, the end-systolic volume was also increased in the IR group and there was a marked beneficial effect of MLC901 to prevent this increase. This beneficial effect was also observed with measurements of the end-systolic area of the left ventricle. Here again, there was a very significant compensative effect of MLC901 to prevent the increase observed in the IR group one month after infarction.

All these results obtained from fibrosis quantification and echocardiography indicate that MLC901 is a potent treatment against post-MI LV remodelling. These data are supported also by the significant increase in the vessel density evaluated after a 4-month treatment in the infarcted area in MLC901 treated compared to non-treated IR hearts, suggesting that MLC901 is able to induce a vascular remodelling and repair as already reported after ischemic stroke in a mouse model of middle cerebral artery occlusion^26^. Our results suggest that MLC901 could stimulate revascularization after myocardial ischemia-reperfusion. Recent developments have shown that alteration of the coronary microcirculation^52^ due to haemorrhage and microvascular obstruction was associated with bad prognosis for AMI patient’s mortality^53^. In addition, following the failure of clinical trials in cardioprotection, recent recommendations from the clinicians have been made to take into account the coronary circulation as a target of cardioprotection^10,42^. MLC901 in this context could reduce mortality rates by decreasing infarct size and inducing vascular remodelling of the infarcted area.

EF was measured from the bidimensional mode (B-mode) after evaluation of the systolo-diastolic cavity volumes and from the area-length method^54^. EF is the main indicator of cardiac performance and remains the simplest and most widely used parameter for the global assessment of LV function. Ejection fraction is a strong predictor of mortality and retains a central role in clinical decisions, including guidelines regarding valve surgery and device implantation^55^.

In our study, the reduction in cardiac injury with MLC901 was paralleled with an improvement in LV systolic performance. MLC901-treated mice showed higher LV ejection fraction (LVEF) from 1 month to 4 months after MI compared to non-treated IR mice. These findings are important because better LVEF is definitely associated with improved outcomes.

An important effect of the MLC901 treatment is that it seems to reduce mortality after myocardial infarction. This positive effect is associated with a trend in the improvement of survival rates in the group of mice treated by MLC901 compared to the non-treated IR during the 4 following months. In our *ex vivo* study, we observed an increase in infarct size for the highest dose tested at 500 ng/ml. However, we did not observe any toxicity for MLC901 at the corresponding dose of 40 µg/kg *in vivo*. This difference could be related to the fact that *ex vivo*, the concentration of MLC901 is artificially maintained constant at this high dose during the perfusion duration, i.e. one hour. This is not the case *in vivo* where MLC901 (intravenous or *per os* administration) diffuses in all the different compartments of the internal environment. Our mouse *in vivo* study corroborates data obtained in clinical trials for MLC601 and MLC901^14,17–20,23,24,28,56–60^.

LV stroke volume is an important index of cardiac performance that has been used to gauge therapeutic response and predict adverse clinical event risk^61^. Stroke volume, an important determinant of cardiac output, was evaluated by echocardiography as the difference between the diastolic and systolic volumes. MLC901 was found to significantly prevent the IR-induced decrease in stroke volume observed at 4 months. MLC901 definitely improved cardiac performance after 4 months of treatment.

Strain echocardiography provides a detailed evaluation of myocardial mechanics. It predicts clinical outcomes better than LVEF in patients with previous MI^62^. In mice, our data clearly indicate that MLC901 treatment during AMI improved 2D longitudinal strain. Changes observed in our study were more consistently significant for global longitudinal strain compared to radial and circumferential strains, in accordance with greater vulnerability of endocardial and subendocardial tissue (longitudinal fibers) to ischemia after coronary occlusion^38^. A recent study performed in mice using STI-methods for echocardiographical analysis of cardiac performance revealed that global longitudinal strain was closely correlated with infarct size and this correlation was better than that between radial strain and infarct size^39^. These observations are related to the clinical situation where longitudinal strain value is considered as an independent predictor of mortality in high-risk patients with AMI^63^. Global longitudinal strain was also reported to be highly sensitive in identifying myocardial dysfunction early in the time course of myocardial infarction and subsequently in the follow-up period with more clear evidence of detrimental effect of AMI^38^.

Measurements of conventional parameters for the study of systolic function (ejection fraction, STI-derived ejection fraction) and also advanced speckle-tracking based strain measures of cardiac performance, in particular global longitudinal strain show that global left ventricular function was improved in the group of mice treated by MLC901 compared to the non-treated IR group.

In conclusion, this paper clearly demonstrates that MLC901 treatment administered early at reperfusion and administrated continuously during 4 months post-infarction provides long-term cardioprotective effects in a murine model of myocardial ischemia-reperfusion. MLC901 treatment is characterized by a reduced cardiac injury, a preserved vessel density in the infarcted area and an improved cardiac performance. These beneficial effects seem to be associated with reduced mortality rates. These results also probably explain the protective cardiovascular effects of the parent TCM compound MLC601 previously observed in stroke patients^17^. MLC901 and MLC601 contain the same combination of plant extracts. Both compounds have been shown to be safe in clinical trials and both provide the same type of neurological recovery after brain injuries in rodents^21,22,25,26^.

In addition to its use for cerebrovascular diseases and particularly for stroke^17–20,23^, results presented here highlight a new potential application of MLC901 as a therapeutic tool against myocardial ischemia-reperfusion injury.

## Methods

All experiments were carried out on C57BL/6J mice (Charles River laboratory, l’Abresle, France) in accordance with the European Communities Council directive of November 1986 and conformed to the “Guide for the Care and Use of Laboratory Animals” published by the US National Institutes of Health (NIH publication 8th Edition, 2011). The surgical protocols were approved by the “Comité d’éthique pour l’expérimentation animale Languedoc-Roussillon” (CEEA-LR) with the authorization number CE-LR-0814.

Expanded methods are described in the Supplementary Information file.

### Surgical protocol of myocardial ischemia/reperfusion

Investigation conforms to the Directive 2010/63/EU of the European Parliament. Acute myocardial ischemia and reperfusion were performed on male mice as described in details in the Supplementary Information file. Various protocols were applied:

- IR: 40 minutes ischemia–60 minutes reperfusion;
- IR_24h_: 40 minutes ischemia–24 hours reperfusion;
- IR_4m_: 50 minutes ischemia–4 months reperfusion;
- SHAM: sham operation without coronary artery ligation (surgery placebo).

### Langendorff *ex vivo* studies

The heart was mounted on a Langendorff system and retrogradely perfused at pressure- and temperature-constant (37 °C) saline solutions. Longitudinal contraction was measured through a small hook attached to the apex of the heart and connected to a force transducer connected via the DMT amplifier. The recordings were analyzed using Clampfit 9.2 sofware (Axon Instruments). Ischemia-reperfusion was achieved by either using global (I_30_R) or regional ischemia models on EMKA system allowing coronary blood flow and ECG recordings.

### Measurement of infarct size

Left ventricles (LV) were sliced transversally into 1-mm-thick sections and incubated in a 1% solution of 2,3,5-triphenyltetrazolium chloride (TTC; *Sigma-Aldrich*). The ischemic risk area and the infarcted area were measured by planimetry with Image J software (Scion Corp).

### DNA fragmentation assay

Specific DNA fragmentation was quantified in transmural samples of non-ischemic or ischemic areas of the LV with an enzyme-linked immunosorbent assay kit (*Roche Diagnostics*) designed to measure the amount of cytosolic oligonucleosome-bound DNA, as previously described^64^.

### Isolation and culture of ventricular cardiomyocytes

Isolated cardiomyocytes were obtained from adult mouse hearts enzymatically dissociated. To do this, hearts were mounted on a Langendorff apparatus and subjected to 1-hour global ischemia followed by 1-hour reperfusion (IR group). At the end of reperfusion, hearts were perfused with an enzymatic solution (liberase™; *Roche Diagnostics*). Single rod-shape myocytes were plated on laminin-coated glass bottom chambers (for Ionoptix experiments).

### Analysis of single myocyte contractility (IonoptixTM system)

The Ionoptix^TM^ system allows to study the contraction of cardiomyocytes in real time *via* measurement of the sarcomere length. Isolated cardiac myocytes were disposed in a low volume chamber and exposed to warm Tyrode solutions.

The Ion Wizard6^TM^ software was used on the Ionoptix system coupled to a MyoCam-S^TM^ camera for the acquisition and the analysis of the data. Solutions of MLC901were perfused during 200 seconds after a stabilization period.

### Blood sample collection

Blood (200 µl) was collected from the retro-orbitary sinus in anesthetised mice (ketamine-xylazine) in heparin tubes at various time points (24 hours and 1 month post-surgery). The blood was centrifuged at 4 °C and supernatants were stored at −80 °C.

### Quantification of plasmatic cardiac troponin I

The amount of plasmatic cardiac troponin I (cTnI) at various time points was quantified with the high sensitivity mouse cardiac Troponin-I Elisa immunoassay kit (*Life Diagnostics*) using a Tecan Infinite M 200 system.

### Pharmacological treatments

MLC901 powder (NurAiDII™; *Moleac*, Singapore) was tested at three doses *in vivo*: 0.4, 4 and 40 mg/kg. For *ex vivo* and *in vitro* experiments, MLC901 was tested at 5, 50 and 500 ng/ml corresponding to the *in vivo* doses taking into account a weight of 25 g *per* mouse.

Drugs were administered *in vivo* intravenously 5 min before reperfusion during the surgical protocol and *ex vivo* in the solution of reperfusion.

For the long-term follow-up, a MLC901 solution at 10 mg/ml was administered *per os* in the drinking water (treatment starting 24 h post-surgery).

### Immunoblotting

Tissue samples (left ventricle) were rapidly frozen in liquid nitrogen after the end of surgery, homogenized with a grinder in RIPA buffer supplemented with EDTA free protease and phosphatase inhibitor. Protein concentrations were determined with the bicinchoninic acid (BCA) protein assay kit (*Pierce*). Samples of protein were resolved by SDS polyacrylamide gel electrophoresis and transferred to nitrocellulose. Anti-AKT, anti-pAKT, anti-ERK1/2, anti-pERK1/2, anti-JNK, anti-pJNK were used. Anti-α actinin antibody was used as internal control for equal loading of total protein extract. Protein bands were visualized by enhanced chemiluminescence method and densitometry analysis was performed using Chemidoc^TM^ (*Biorad*) and ImageJ software.

### Echocardiography

Anaesthetized mice (1–1.2% isoflurane) underwent transthoracic echocardiographic examination at 1, 2, and 4 months after coronary artery ligation as previously described^8^. Cardiac morphology and function were assessed using a high-frequency, high- resolution echocardiographic system consisting of a VEVO ultrasound machine (2100 or 1100) equipped with a 22–55 MHz bifrequencial transducer (*VisualSonics B.V*., The Netherlands). High-resolution images were obtained in the parasternal long and short axes, apical and supra-sternal orientations.

Using the Vevostrain software and the speckle tracking imaging, global longitudinal and radial strains were measured from the bidimensional long axis view of the left ventricle. Circumferential and radial strains were measured from the bidimensional short axis view.

### Fibrosis and vascular density measurement

LV tissue specimens were fixated in 4%-PFA and embedded in paraffin, cut from the apex through the base and stained with Picrosirius red. Slides were scanned to acquire whole slide imaging at × 20 magnification. Total fibrosis area was reported to the total area for each slice.

For the detection of capillary vessels, Isolectin B4 FITC Conjugate (*Sigma-Aldrich*) staining was performed on paraffin-embedded LV sections of the middle part of the LV. Nuclei were stained with DAPI (*Sigma-Aldrich*). Capillary density was calculated in a blind fashion by two experimenters as the average number of IB4 + vessels *per* mm^2^. For counting, n = 3 areas were randomly taken both in the border zone of the infarcted remote areas and in the remote zone of one section *per* left ventricle. Images obtained with a Zeiss *Axioimager Z3* fluorescent microscope were analyzed using ImageJ.

### Statistical analysis

Data were expressed as mean ± standard deviation. The statistical analysis was performed using GraphPad Prism (*GraphPad Software, San Diego, USA*).

One-way ANOVA for parameters measured at one time point and two-way ANOVA for parameters measured at multiple time points (repeated measures) were used. A Tukey’s post-test for multiple comparisons was applied when appropriate. When only two groups were compared, the Mann-Whitney test was used.

For Kaplan-Meier analysis, comparison of the curves was performed using the Logrank (Mantel-Cox) test. Statistical significance was noted as ns for p > 0.05,* for p < 0.05, ** for p < 0.01, *** for p < 0.001 and **** for p < 0.0001.

### Data availability statement

Materials, data and associated protocols are available to readers.

## Electronic supplementary material

supplementary information
