# Supplementary material for: Acute and long-term cardioprotective effects of the Traditional Chinese Medicine MLC901 against myocardial ischemia-reperfusion injury in mice
Source: Sci Rep. 2017 Oct 31;7:14701. doi: 10.1038/s41598-017-14822-x (PMC5665902; doi:10.1038/s41598-017-14822-x)
Supplement: Supplementary file 1 — supplementary information [file 41598_2017_14822_MOESM1_ESM.pdf]

Acute and long-term cardioprotective effects of the Traditional Chinese Medicine MLC901 against myocardial ischemia-reperfusion injury in mice

By A. Vincent, A. Covinhes, C. Barrère, L. Gallot, S. Thoumala, C. Piot, C. Heurteaux, M. Lazdunski, J. Nargeot, S. Barrère-Lemaire.

Supplementary information

Expanded methods

Study design

In this study, various durations of ischemia have been used: 30, 40 or 50 minutes depending on the protocol. These protocols were chosen in order to evaluate in the most appropriate manner the cardioprotective properties of MLC901. Most of the experiments *in vivo* were performed using the $I_{40}R_{60}$ protocol with coronary ligation (40 minutes of ischemia, followed by 60 minutes of reperfusion) as reported in our previous *in vivo* studies [1-5]. It is also important to note that the *ex vivo* study on the EMKA Langendorff system performed on isolated hearts with a protocol of 40 minutes ischemia followed by 60 minutes of reperfusion allowed providing the same injury as *in vivo* with the same protocol. Unlike in our previously published *in vivo* studies, *in vivo* surgery for this paper was realized in a brand new animal facility where only *Specific Pathogen-Free* (SPF) animals under controlled sanitary conditions were used. Probably because of the reduction of environmental stresses, we observed no mortality during the post-infarction period for a long-term follow-up, showing clearly that the injury was less severe than that obtained in our "old" surgical room. It has been published in the literature and reported by many researchers that the laboratory environment may influence results [6]. Then to increase the injury, the ischemia duration was prolonged from 40 to 50 minutes in the long-term study. This protocol allowed recovering mortality rates described by other groups for the post-infarction period [7]. For *ex vivo* global ischemia experiments (no-flow system instead of coronary artery ligation), 40 minutes of global ischemia was initially applied but the injury was too strong (infarct size was 90% of the left ventricle). Then, the ischemia duration was decreased from 40 to 30 minutes ($I_{30}R_{60}$ protocol). It is important to underline that these changes in ischemia duration were performed to improve the quality control of the experiments.

Surgical preparation of myocardial ischemia-reperfusion (IR)

All experiments were carried out on C57BL/6J mice (Charles River laboratory, l'Abresle, France) in accordance with the European Communities Council directive of November 1986 and conformed to the Guide for the Care and Use of Laboratory Animals" published by the US National Institutes of

Health (NIH publication 8th Edition, 2011). The surgical protocols were approved by the "Comité d'éthique pour l'expérimentation animale Languedoc-Roussillon" (CEEA-LR) with the authorization number CE-LR-0814.

Acute myocardial ischemia and reperfusion were performed in C57Bl6 mice as previously described[3,5]. Male mice (22-30 g) were anaesthetized with an intramuscular (IM) injection of an anaesthetic mixture comprising ketamine (50 mg/kg; Imalgène® 500; *Merial, France*), xylazine (10 mg/kg; Rompun® 2%; *Bayer, France*) and chlorpromazine (1.25 mg/kg; Largactil® 5 mg/ml; *Sanofi-aventis, France*). Mice were ventilated via a tracheal intubation on a Harvard rodent respirator (tidal volume 7.2 µl/g, body mass; respiratory rate 200 breaths per min). The body temperature was maintained constant between 36.8 and 37° C owing to the thermo-regulated surgical table connected to a rectal probe. After a second injection of ketamine (50 mg/kg) and xylazine (10 mg/kg), the chest was opened by a left lateral thoracotomy and a reversible coronary artery snare occluder was placed around the left coronary artery. The adequacy of anesthesia and pain analgesia was monitored during the surgical protocol by pinching the end of the posterior legs. All animals underwent an ischemic insult of 40 min. Reperfusion was achieved by loosening the knot.

- For the protocol IR (40 min ischemia-60 min reperfusion), a third administration of ketamine/xylazine was performed (same dose as in the previous injection, IM) at the onset of reperfusion. The artery was re-occluded. The phtalocyanine blue dye was injected into the left ventricle (LV) cavity and allowed to perfuse the non-ischemic portions of the myocardium. Hearts were harvested leading to euthanasia. The left ventricles were dissected out (atria and right ventricle removed) and dedicated to infarct size or DNA fragmentation measurements.
- When reperfusion lasted more than 60 min (24h or 4 months), mice underwent a recovery protocol comprising subcutaneous administration of lidocaine (1.5 mg/kg; Xylocaïne® 10 mg/ml; *AstraZeneca, France*) and a postoperative awakening in an emergency care unit maintained at 28°C, with oxygen supply and controlled humidity. After a period of 2 hours stabilization, mice were allowed to recover in a ventilated cabinet during 20 hours.

- For the protocol IR_{24h} (40 min Ischemia-24h reperfusion), mice were re-anesthetized 24 hours after the onset or reperfusion with an intramuscular (IM) injection of an anaesthetic mixture comprising ketamine (50 mg/kg; Imalgène® 500; *Merial, France*), xylazine (10 mg/kg; Rompun® 2%; *Bayer, France*) and chlorpromazine (1.25 mg/kg; Largactil® 5 mg/ml; *Sanofi-aventis, France*). Mice were ventilated *via* a tracheal intubation on a Harvard rodent respirator (tidal volume 7.2 µl/g, body mass; respiratory rate 200 breaths per min). The chest was opened by cutting the sutures performed the day before. The artery was re-occluded thanks to the prolene link kept in place into the chest, the phtalocyanine blue dye injected into the left ventricle (LV) cavity and the left ventricle dissected out (atria and right ventricle removed) and dedicated to infarct size or DNA fragmentation measurements.

- For the protocol IR_{4m} (50 minutes ischemia - 4 months reperfusion), mice were subjected to a 4-month follow-up program comprising an echocardiographic evaluation of their functional post infarction recovery at 1, 2 and 4 months after the surgery. After the last echocardiographical evaluation, mice were sacrificed by cervical dislocation under anesthesia induced by intramuscular injection of an anaesthetic mixture comprising ketamine (50 mg/kg; Imalgène® 500; *Merial, France*), xylazine (10 mg/kg; Rompun® 2%; *Bayer, France*) and chlorpromazine (1.25 mg/kg; Largactil® 5 mg/ml; *Sanofi-aventis, France*). Hearts were harvested and dedicated to picrosirius red staining for fibrosis determination and immunochemistry analysis.

The sham-operated group (SHAM): the mice underwent sham operation without coronary artery ligation.

Langendorff *ex vivo* studies

For *ex vivo* experiments, C57Bl6 male mice were anesthetized with a first intramuscular injection of an anesthetic cocktail comprising ketamine (0.12 mg/ml, Imalgène®, Merial) and xylazine (0.8 mg/ml, Rompun®, Bayer), and by a second injection of 0.3 ml of pentobarbital (0.6 mg/ml, Sanofi). Mice were treated (IP injection) by heparine (0.5 ml) in order to prevent the blood clot formation. The heart was excised after sternotomy and cannulated through the ascending aorta.

Then, the heart was quickly mounted on the Langendorff system and retrogradely perfused at pressure- and temperature-constant by pre-heated Krebs solution (37°C). The Krebs solution (NaCl 116 mM ; KCl 5 mM ; $MgSO_4$ $7H_2O$ 1,1 mM ; NaH_2PO_4 0.35 mM ; $NaHCO_3$ 27 mM ; Glucose 10 mM ; $CaCl_2$ 1.8 mM ; pH 7.4) was oxygenated with carbogen. In order to evaluate cardiac contraction amplitude *ex vivo*, the heart was mounted on a modified Langendorff system (allowing a retrograde perfusion of the aorta) and bathed in a warm chamber containing oxygenated Krebs solution. Longitudinal contraction was measured through a small hook attached to the apex of the heart and connected to a force transducer connected *via* the DMT amplifier. The recordings were obtained on a PC computer through a digidata and PClamp 9.2. All the results have been analyzed using Clampfit 9.2 sofware (Axon Instruments).

Ischemia-reperfusion was achieved *ex vivo* by either using global ($I_{30}R$) or regional ischemia (IR) models. Global ischemia was induced by stopping the perfusion during 30 minutes (no-flow). Reperfusion is achieved by restoring the liquid perfusion during 60 minutes with the Krebs solution (control) or with MLC901. Regional ischemia-reperfusion model was obtained using an EMKA system allowing coronary blood flow and ECG recordings. The coronary circulation was perfused at 70 mmHg over a 1-4 ml/min flow range with a gassed modified Krebs-Henseleit buffer. During the stabilization phase, a 7.0 prolene ligature (Johnson & Johnson Ethicon, Belgium) was placed around the left coronary artery 1-2 mm distal from where it emerges beneath the left atrium and the ends of the ligature were passed through an occluder tube, initially without tension. Heart temperature was continuously assessed by a probe positioned on the base of the heart and recorded by using a TH-5 monitoring thermometer (Phymep, USA). Perfused hearts were then immersed in a water-jacketed bath and continuously maintained at 37°C. After a stabilization phase of 15 min (baseline), regional normothermic ischemia was then induced by tightening the suture with the occluder tube for 40 min. After ischemia, reperfusion was allowed for 1 hour by loosening the ligature. At the end of reperfusion, the artery was reoccluded, the hearts were removed from the perfusion system and phthalocyanine blue was injected through the aortic cannula and allowed to perfuse the non-ischemic portions of the myocardium, making possible to delineate the area at risk.

Recording of coronary blood flow

During the whole *ex vivo* IR protocol, the coronary flow was continuously digitized at 0.1 Hz and recorded using an IOX data acquisition system (EMKA Technologies, France). Excel software (Microsoft, USA) was used to perform offline analysis of the data recorded. For each mouse, a mean value of maximal coronary flow was calculated each 10 minutes. Values during ischemia and reperfusion were normalized with respect to the pre-ischemia phase (baseline) and were expressed in percentages.

ECG recordings and analysis.

ECG was continuously recorded by Ag/AgCl electrodes positioned on the right atrium and near the apex.

Signals were digitized continuously at 1 kHz and recorded using an IOX data acquisition system (EMKA Technologies, France). The software ecgAUTO (EMKA Technologies, France) was used to perform offline analysis of the data recorded. For each mouse the mean HR value during baseline, ischemia and reperfusion was calculated.

Measurement of infarct size

At the end of the perfusion protocol, the right ventricle and the atrias were quickly removed. The left ventricle was included in a 4% Agar block and cut in 1mm-thick slices. Infarct size measurements were obtained after coloration of left ventricular sections with 2,3,5-triphenyltetrazolium chloride (TTC; *Sigma-Aldrich*) and were expressed as a percentage of the ischemic risk area. Ischemic area was delineated by phtalocyanin blue dye coloration in case of coronary ligature (regional ischemia). Then the slices were fixed in a 4%-paraformaldehyde (in PBS solution) during 48 h. Each slice was photographed on each side thereof. The extent of infarction area (not stained in brick-red) was measured by planimetry using the ImageJ software. Infarct size was expressed as a percentage of LV mass.

DNA fragmentation assay

Specific DNA fragmentation was quantified in transmural samples of non-ischemic or ischemic areas of the LV with an enzyme-linked immunosorbent assay kit (*Roche Diagnostics*) designed to measure the amount of cytosolic oligonucleosome-bound DNA, as previously describe[8]. Transmural samples from 30 mg of non-ischemic areas of the left ventricle (central portion of the septum) and ischemic areas (portion of the LV free wall directly under the silk ligation) were harvested on mice submitted to the surgical protocol. Tissues samples were disintegrated in a tissue grinder in 400 µl of lysis buffer supplied with the kit. The homogenate was centrifuged at 13,000 g for 10 min. The supernatant was used as antigen source in the sandwich ELISA. Incubation buffer, instead of the sample solution, and DNA-histone complex were used as negative and positive controls, respectively. Two values from the double absorbance measurements (405 nm / 490 nm) of the samples were averaged, and the background (negative control) was subtracted from each of these averages. The positive control was used as an internal control for daily variability. DNA soluble nucleosome was quantified both in blue-colored region (non-ischemic region = NI) as well as in the non blue-colored areas (ischemic region = I). This allows to normalize the rate of DNA fragmentation (internal control) and to avoid the introduction of variability in the results due to differences in temperature, timing of the measurement.

Isolation and culture of ventricular cardiomyocytes

Isolated cardiomyocytes were obtained from adult mouse hearts enzymatically dissociated. To do this, hearts were mounted on a Langendorff apparatus and subjected to 1hour-global ischemia followed by 1hour-reperfusion (IR group). At the end of reperfusion, hearts were perfused with an enzymatic solution (liberase™; Roche). Single rod-shape myocytes were plated on laminin-coated glass bottom chambers (for Ionoptix experiments).

Analysis of single myocyte contractility (Ionoptix™ system)

The Ionoptix™ system allows to study the contraction of cardiomyocytes in real time *via* measurement of the sarcomere length. Isolated cardiac myocytes were disposed in a low volume chamber (500 µl) and exposed to warm Tyrode perfusions by electronic valves (Warner Corp.).

The Ion Wizard6™ software was used on the Ionoptix system coupled to a MyoCam-S™ camera for the acquisition and the analysis of the data. The SarcLen™ mode provides analysis of the sarcomere shortening (see Figure 4). In brief, cardiomyocytes were electrically stimulated at 1.5 Hz until contractions became uniform. The averaged percentage of sarcomere shortening was obtained from10 to 20 consecutive contractions. Solutions of MLC901were perfused during 200 seconds after a stabilization period of Tyrode perfusion (200 sec. also).

Pharmacological treatments

NurAiDII™ (MLC901) was provided by Moleac (Singapore). A capsule containing 400 mg MLC901 was diluted in 40 ml NaCl 0.9% medium corresponding to a concentration of 10 mg/ml (stock solution) at 37°C during 60 min. The solution was then filtered with 0.22 µm filter. The stock solution (10 mg/ml) was stored away from light at 4°C (maximum of 4 days).

The dosage used in this work has been determined according to the formula currently used for dose translation from animal to human studies[9]. For *in vivo* MLC901 injection in mice, the authors have adapted the concentrations previously used for intraperitoneal injection in neuroprotection studies[10]: the 40 µg/kg IP dose (2 µg/ml MLC901 solution diluted in saline (as vehicle) in a total volume of 500 µl/mouse weighing 25 g) was divided ten times for IV injection: 4 µg/kg.

For the *per os* administration: considering that a mouse drinks 5 ml per day and weights 25 g, the MLC901 dose ingested by mice at the concentration of 6 g/l was around 30 mg/day, which corresponds to a HED of 97 mg/kg and to the dose used in humans for clinical trials (4 capsules, oral administration, three times a day) [11]. For the *in vivo* experiments, MLC901 was tested at three doses: 0.4, 4 and 40 mg/kg. For *ex vivo* and *in vitro* experiments, MLC901 was tested at the same doses taking into account a weight of 25 g *per* mouse (the corresponding solution concentration used for perfusion were 5, 50 and 500 ng/ml, respectively).

Drugs were administered *in vivo* intravenously 5 min before reperfusion during the surgical protocol and *ex vivo* in the solution of reperfusion.

For the long-term follow-up, a MLC901 solution at 10 mg/ml was prepared every 3 days and was administered *per os* in the drinking water (treatment starting 24h post-surgery).

Immunoblotting

Tissue samples (left ventricle) were rapidly frozen in liquid nitrogen after the end of surgery. Samples were homogenized with a grinder in RIPA buffer supplemented with EDTA free protease and phosphatase inhibitor (Halt™ Protease and phosphatase single-use inhibitor Cocktail-100X, *Thermoscientific*). After centrifugation, pellets were resuspended in RIPA buffer for protein purification and incubated 1 h at 4°C. Protein concentrations were determined with the bicinchoninic acid (BCA) protein assay kit (*Pierce*). Samples (25 µg) of protein were resolved by SDS polyacrylamide gel electrophoresis (4-20% mini-Protean®TGX™ precast gels; *Biorad*) and transferred to nitrocellulose (Trans-Blot®Turbo™; *Biorad*). The following antibodies were used: anti-AKT, anti-pAKT, anti-ERK1/2, anti-pERK1/2, anti-JNK, anti-pJNK (*Cell Signaling Technology®)*. Anti-α actinin antibody (*Cell Signaling Technology®)* was used as internal control for equal loading of total protein extract. After this, membranes were incubated with the secondary antibody (Donkey anti Rabbit Ig-G HRP, *Jackson ImmunoResearch laboratories*) for 1 h at room temperature and then washed twice with TBST for 15 min. Protein bands were visualized by enhanced chemiluminescence method using ECL kit (SuperSignal™ West Pico chemiluminescent Substrate, *Thermo Scientific™*). Densitometry analysis was performed using Chemidoc™ (*Biorad*) and ImageJ software.

Blood sample collection

Blood (200 µl) was collected from the retro-orbitary sinus in anesthetised mice (ketamine-xylazine) in heparin tubes at various time points (24 hours and 1 month post-surgery). The blood was centrifuged at 4°C during 10 minutes at 1,100 g and supernatants were stored at -80°C.

Quantification of plasmatic cardiac troponin I

The amount of plasmatic cardiac troponin I (cTnI) at various time points was quantified with the high sensitivity mouse cardiac Troponin-I Elisa immunoassay kit (*Life Diagnostics*) using a Tecan Infinite M 200 system. The immunoassay detects murine cTnI with an analytical measuring range between 10-0.156 ng/ml. The assay was performed in duplicates.

Echocardiography

Mice underwent transthoracic echocardiographic examination, 1, 2, and 4 months after the coronary artery ligation as previously described[5]. *In vivo* cardiac morphology and function were assessed noninvasively using a high-frequency, high- resolution echocardiographic system consisting of a VEVO ultrasound machine (2100 or 1100) equipped with a 22–55 MHz (MS-550D) bifrequencial transducer (VisualSonics B.V., The Netherlands). Mice were anaesthetized with 1-1.2 % isoflurane for induction and were placed in supine position on an imaging stage equipped with built-in electrocardiographic electrodes for continuous heart rate monitoring and a heater to maintain the body temperature at 37°C. High-resolution images were obtained in the parasternal long and short axes, apical and supra-sternal orientations. Standard B-mode images of the heart and pulsed wave Doppler images of the mitral and aortic inflows were acquired. LV dimensions and wall thickness were measured at the level of the papillary muscles in short axis and in long axis views during the end systole and end diastole. LV ejection fraction (EF), fractional shortening (FS - longitudinal shortening), and mass were calculated. All the measurements and calculations were done in accordance with the American Society of Echocardiography. In order to better evaluate echocardiographic parameters after myocardial infarction, B-mode views were acquired at end-diastole and end-systole. In particular, B-mode measurements were based on endocardial outlining on both parasternal long axis (PLAX) and short axis (PSAX) by Vevolab software (LV trace) and Vevostrain software (2D-strain).

Two-dimensional strain (2D-strain) is a method of quantifying regional tissue deformation from continuous frame-by-frame tracking of acoustic speckles (speckle tracking imaging = STI), which is angle independent and less subject to artefact[12]. Global LV function was assessed either from averaging of segmental 2D-strain and from automated measurement of EF obtained from endocardial detection in end-diastole and end-systole from speckle tracking.

LV end-diastole area (area;d), LV end-systole area (area;s). Based on these parameters, end diastolic and end systolic LV volumes (volume;d and volume;s), ejection fraction (EF,%), fractional shortening (FS - longitudinal shortening), stroke volume (SV) were calculated. Using the Vevostrain software and the speckle tracking imaging, global longitudinal and radial strains were measured from the

bidimensional long axis view of the left ventricle. Circumferential and radial strains were measured from the bidimensional short axis view. Speckle tracking imaging-derived ejection fraction (EF) and fractional area change (FAC) were calculated from these measures (See **Table S1** for all values).

Euthanasia and organ harvesting

4 months after the surgery, mice were anesthetized by injecting a mixture of ketamine (50 mg/kg; Imalgène® 500), xylazine (10 mg/kg; Rompun® 2%). The chest was opened and the heart was harvested (leading to the sacrifice of the animal) and rinsed in a PBS saline solution. The right ventricle and the atrias were removed and the left ventricle was fixated in a 4%-PFA solution during 48 hours.

Fibrosis and vascular density measurement

Left ventricles tissue specimens obtained from SHAM, IR and MLC901 treated mice were fixated in 4%-PFA and embedded in paraffin. Each left ventricle was cut from the apex through the base (4 µm sections each 300 µm) and stained with Picrosirius red according the protocol designed by the RHEM platform (Biocampus, Montpellier). The stained slides were scanned on a whole slide scanner (Nanozoomer 2.0-HT, *Hamamatsu*) to acquire whole slide imaging at ×20 magnification. Fibrosis revealed by Picrosirius staining was quantified by measuring the extent of fibrotic area in each heart cross-sections using an *ImageJ* macro developed by MRI platform (Montpellier). Total fibrosis area was reported to the total area for each slice. The amount of total fibrosis in each LV was obtained by averaging the % of fibrosis of all the slices across the whole LV. Slides, in particular those including a majority of non-myocardial fibrosis, i.e. valvular, were excluded from the calculation. In the SHAM group, a second fibrosis evaluation was performed by excluding the perimeter fibrosis (probably fibrosis of adherence) resulting from post-surgery remodeling of the heart in the chest (absence of pericardium). Thereafter, the mean value of the percentage corresponding to the fibrosis of adherence was deduced from the fibrosis values (in %) of each left ventricle from mice of the IR and MLC901 groups.

For the detection of capillary vessels (in particular endothelial cells), Isolectin B4 FITC Conjugate (*Sigma-Aldrich*) staining was performed on paraffin-embedded LV sections (4 µm) of the middle part of the LV. Nuclei were stained with DAPI (*Sigma-Aldrich*). Capillary density was calculated in a blind fashion by two experimenters as the average number of IB4+ vessels per mm^2, using a fixed frame of 665.6 x 665.6 μm^2. The number of positive IB4 staining was counted in 6 fields per section (3 in the ischemic area and 3 in the remote non-ischemic region). Stained sections were mounted in Fluoroshield™ (*Sigma-Aldrich*). Images were obtained with a Zeiss *Axioimager Z3* fluorescent microscope and analyzed using ImageJ and Adobe Photoshop.

Statistical analysis

Data expressed as mean ± standard deviation (rounded values two digits after the decimal point). The statistical analysis was performed using GraphPad Prism (*GraphPad Software, San Diego, USA*).

One-way ANOVA with Tukey's post-test for multiple comparison for parameters measured at one time point and two-way ANOVA with Tukey's post-test for multiple comparisons for parameters measured at multiple time points (repeated measures). When only two groups were compared, the Mann-Whitney test was used.

For Kaplan-Meier analysis, comparison of the curves was performed using the Logrank (Mantel-Cox) test. Statistical significance was noted ns pour $p > 0.05$,* for $p<0.05$, ** for $p<0.01$, *** for $p<0.001$ and **** for $p <0.0001$.

Supplementary results

Acute effects and safety

Negative inotropism was already reported to protect the heart during myocardial infarction. In this context, the effects of MLC901 on cardiac contraction were tested *ex vivo* and *in vitro*. For these experiments, MLC901 was tested at 5, 50 and 500 ng/ml corresponding to the *in vivo* doses (0.4, 4 and 40 µg/kg, respectively).

Isolated mouse hearts were mounted placed on a modified Langendorff system with spontaneous sinus-driven beating heart and contraction was measured using a DMT® myograph. Hearts were perfused with MLC901 as cumulative doses (**Fig.S1A)**. Neither the heart rate nor the contraction amplitude were modified at any doses tested (**Figs.S1B,C**). This was confirmed *in vitro* on stimulated ventricular cardiomyocytes (**Fig.S2A**) in which the contraction evaluated by the sarcomere length was unchanged upon various concentrations of MLC901 (**Figs.S2B,C**).

Echocardiographic evaluation of systolic performance

Fractional shortening was also evaluated from the bidimensional images using LV-trace. The treatment by MLC901 was able to prevent IR-induced decrease during the whole period of follow-up ($p_{interaction}$ ns= 0.737; $p_{treatment}$ ** = 0.002 and p_{time} ns=0.21). (**Fig.S4A**).

This increased cardiac performance upon MLC901 treatment was also evidenced for the evaluation of the stroke volume, which is an important determinant of cardiac output. The treatment by MLC901 was able to prevent IR-induced decrease during the whole period of follow-up ($p_{interaction}$ ns= 0.09; $p_{treatment}$ ** = 0.002 and p_{time} ns=0.46). (**Fig.S4B**).

Additional Figures

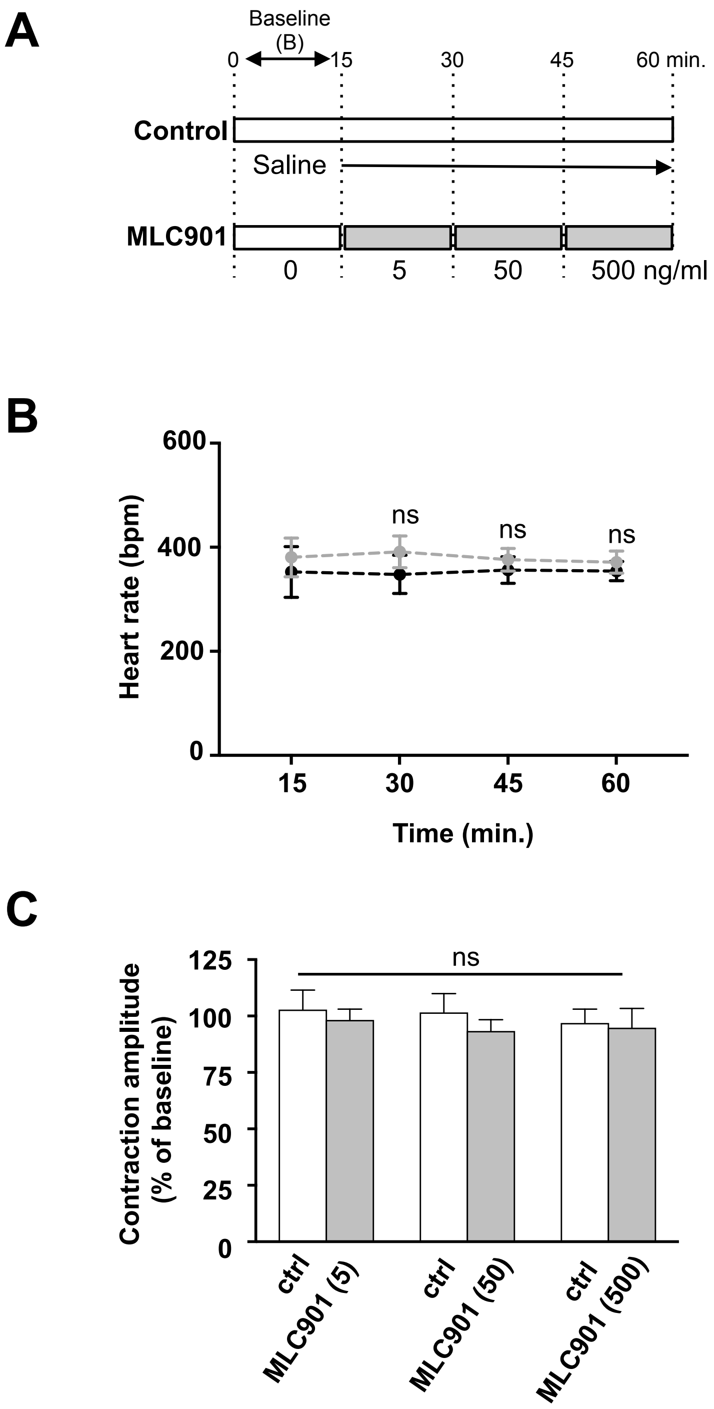

Figure S1: Effect of MLC901 on spontaneous contraction parameters *ex vivo*.

(A): C57Bl6 mouse hearts were mounted on a Langendorff system coupled to a DMT myograph. After a 15 minutes period of stabilization (oxygenated Krebs), hearts were perfused with MLC901 which was administered at 3 different concentrations (cumulative doses) applied during 15 minutes: 5

ng/ml (from 15 to 30 min) ; 50 ng/ml (from 30 to 45 min) and 500 ng/ml (from 45 to 60 min). For the untreated group (black symbols and line), the perfusion was performed with oxygenated Krebs during 60 minutes. For both groups, control (n=6) and MLC901 (n=6), the first 15 minutes correspond to the stabilization period (baseline). Please note that the control group aimed to study the evolution of preparation during 60 minutes. (**B**)**:** Heart rate (in bpm) was measured and (means ± SD) for each period of treatment were plotted. **(C):** Longitudinal contraction was recorded with the DMT myograph and expressed as a % of the initial contraction amplitude measured during stabilization. Parameters of contraction were measured at the end of each period (control or treatment). Histograms represent the mean of only the ten last contractions (mean ± SD). Statistical analysis was performed using two-way ANOVA and a post-test of Sidak for multiple comparisons. Statistical significance was noted ns for $p>0.05$.

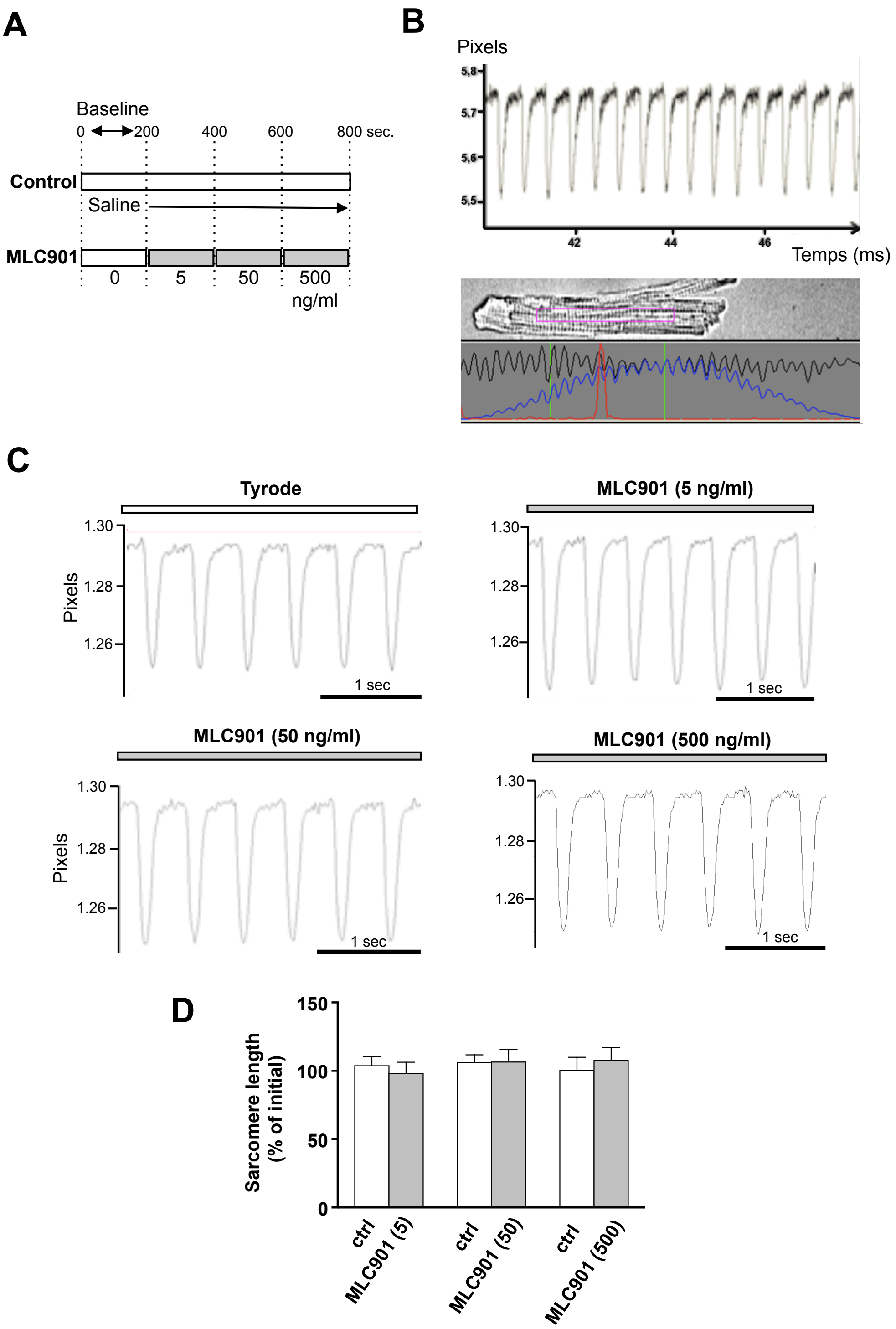

Figure S2: Effect of MLC901 on isolated cardiomyocytes *in vitro*.

(A): Experimental design for evaluation of MLC901 effects on sarcomere length. After a 200 seconds period of stabilization, isolated cardiomyocytes were perfused with 3 different solutions of MLC901 (cumulative doses) applied during 200 seconds in each condition: 5 ng/ml (from 200 to 400 sec.); 50 ng/ml (from 400 to 600 sec.) and 500 ng/ml (from 600 to 800 sec.). For the control condition, Tyrode

was perfused during 800 sec. For both groups, control and MLC901, the first 15 minutes correspond to the stabilization period (baseline). Please note that the control group aimed to study the stability of preparation during 800 sec. **(B):** SarcLen Video image (top) and density trace (bottom, black) obtained during Ionoptix recordings. The Hamming Window (bottom, blue), FFT (Fourier Transform) Power spectrum (bottom, red) and power spectrum thresholds (bottom, green) are presented on the bottom panel. All the calculations were performed on a density trace derived from the region of interest (ROI, pink). To each pixel in the image is assigned a value to indicate it brightness, from 0, black, to 256, white. Plotting the pixel values from a horizontal line in the image gives the density trace. The density trace was used to calculate the average frequency of the sine wave, which can then be converted to wave length (sarcomere length). **(C):** Representative recordings obtained with the Ionoptics system in isolated ventricular cardiomyocytes upon various concentrations of MLC901 (5, 50 and 500 ng/ml). **(D):** Contraction was reflected by variation of the sarcomere length expressed as the % of the initial value measured during stabilization (Baseline). All the sarcomere lengths were analyzed during each period to obtain an average value in each condition. Statistical analysis was performed using two-way ANOVA and a post-test of Bonferroni for multiple comparisons. Statistical significance was noted ns for $p>0.5$. Datas were obtained on $n=8$ cardiomyocytes obtained from 3 independent dissociated heart for each condition.

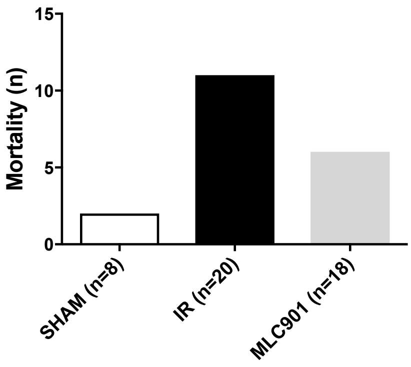

Figure S3: Determination of the mortality rate.

Histogram represents the number of dead mice reported for each group during the 4-month period.

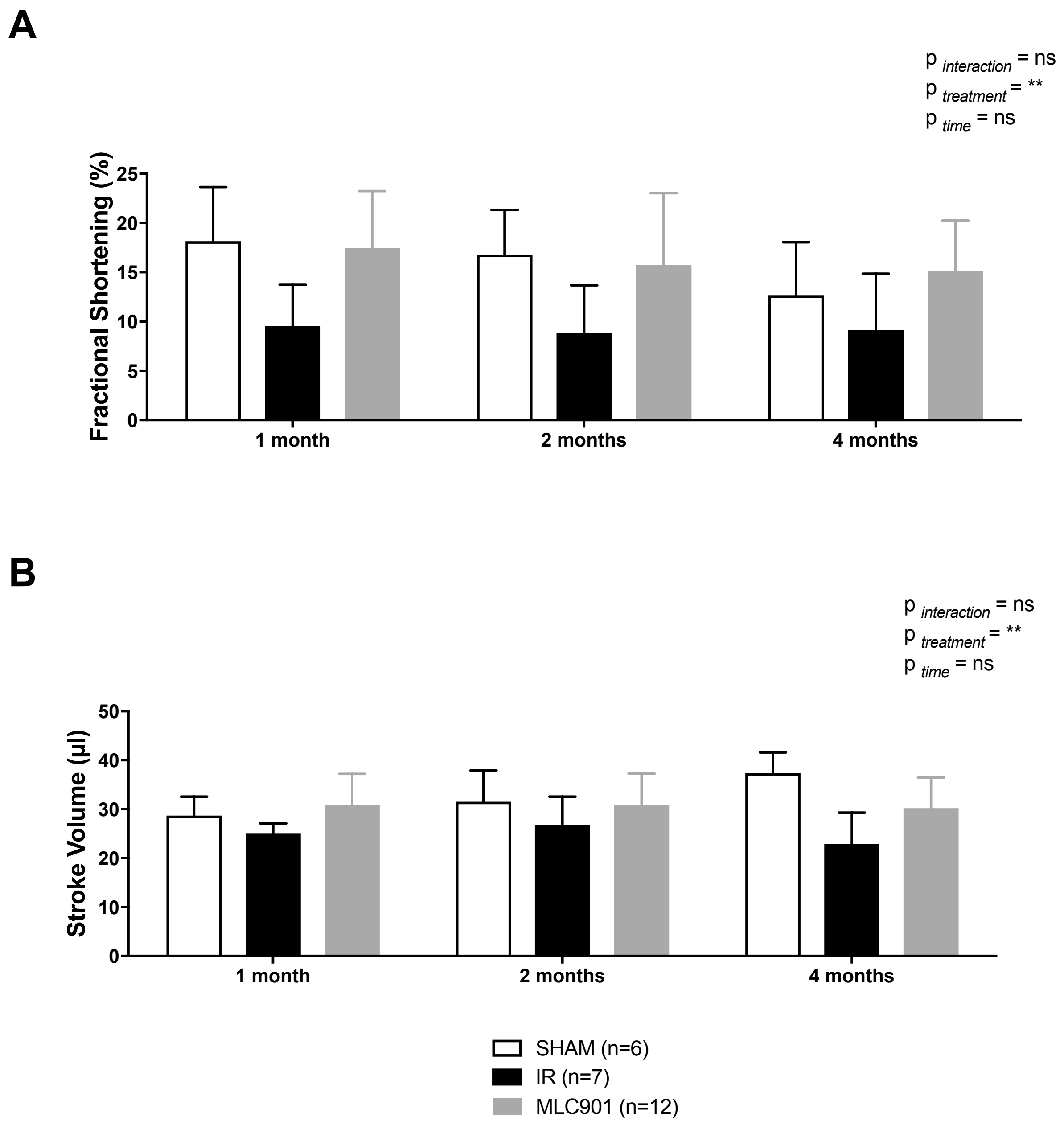

Figure S4: Conventional parameters evaluated by echocardiography (B-Mode). (**A**,**B**): Fractional shortening (FS%) and stroke volume (SV) were calculated from measurements performed on B-mode image of the parasternal long axis (PLAX) view. Data are presented as mean ± SD calculated at 1, 2 and 4 months for FS (**A**) and SV (**B**). Statistical analysis was performed using Two-way ANOVA (treatment and time defined as two parameters) with the Tukey's *post hoc* test for multiple comparisons. Statistical significance was noted ns for $p>0.5$, ** for $p<0.01$.

Supplementary table

		SHAM			IR			MLC901			Two-way ANOVA
		Mean	SD	n	Mean	SD	n	Mean	SD	n	
**Heart rate (bpm)-PLAX**	1 month	531,29	38,58	6	495,20	54,33	7	545,07	43,71	12	P interaction = ns
	2 months	483,09	35,87		463,53	42,81		503,81	31,56		P treatment = *
	4 months	481,95	46,61		515,06	29,94		530,80	48,06		p time = **
**Area;s (mm^2) -PLAX**	1 month	23,69	10,62	6	36,04	3,10	7	23,12	6,07	12	P interaction = ns
	2 months	27,79	7,08		36,09	5,98		29,58	13,49		P treatment = *
	4 months	37,00	7,90		39,98	9,75		32,58	10,95		p time = *
**Area;d (mm^2) -PLAX**	1 month	52,38	11,24	6	50,55	17,76	7	54,02	11,25	12	P interaction = ns
	2 months	59,34	11,47		50,86	20,30		60,47	13,88		P treatment = ns
	4 months	74,37	9,80		52,38	24,33		62,77	16,08		p time = *
**Volume;s (µl) -PLAX**	1 month	13,13	3,73	6	17,31	0,88	7	12,84	2,20	12	P interaction = ns
	2 months	14,56	2,10		16,99	1,48		14,75	3,89		P treatment = *
	4 months	17,43	2,56		17,66	3,03		15,40	3,33		p time = **
**Volume;d (µl) -PLAX**	1 month	21,78	3,02	6	23,74	0,90	7	21,55	2,72	12	P interaction = ns
	2 months	23,44	2,66		23,49	2,06		23,18	2,98		P treatment = ns
	4 months	26,45	2,08		23,27	3,48		23,37	3,59		p time = **
**SV (µl) -PLAX**	1 month	28,69	3,86	6	25,01	2,11	7	30,91	6,29	12	P interaction = ns
	2 months	31,55	6,37		26,69	5,88		30,88	6,38		P treatment = **
	4 months	37,36	4,25		22,92	6,39		30,19	6,29		p time = ns
**EF (%) -PLAX**	1 month	56,65	12,82	6	40,99	2,89	7	57,43	5,21	12	P interaction = ns
	2 months	53,43	6,65		42,40	6,68		52,38	11,95		P treatment = ****
	4 months	50,57	5,51		36,62	5,70		49,08	6,58		p time = *
**FS (%)-PLAX**	1 month	18,15	5,50	6	9,55	4,17	7	17,45	5,79	12	P interaction = ns
	2 months	16,80	4,51		8,88	4,79		15,72	7,29		P treatment = **
	4 months	12,68	5,35		9,14	5,72		15,14	5,10		p time = ns
**Global Radial Strain (%)-PLAX**	1 month	39,59	13,19	5	24,65	11,49	7	34,90	10,53	10	P interaction = ns
	2 months	36,77	9,69		23,39	7,00		29,36	7,52		P treatment = **
	4 months	29,01	3,50		25,74	7,70		34,49	8,60		p time = ns
**Global Long. Strain (%)-PLAX**	1 month	-16,21	3,31	5	-11,34	2,63	7	-19,27	3,72	10	P interaction = ns
	2 months	-15,36	0,85		-13,85	4,93		-17,31	3,95		P treatment = ***
	4 months	-18,59	3,62		-12,47	3,19		-18,64	3,80		p time = ns
**Global Radial Strain (%)-(PSAX)**	1 month	32,24	9,37	6	25,85	10,89	7	37,66	11,66	12	P interaction = ns
	2 months	30,35	10,72		26,44	8,76		30,65	6,45		P treatment = *
	4 months	34,95	4,43		23,56	6,65		36,83	8,80		p time = ns
**Global Circ. Strain (%) -(PSAX)**	1 month	-20,31	3,67	6	-16,77	5,26	7	-21,97	4,16	12	P interaction = ns
	2 months	-19,87	3,75		-17,93	4,41		-17,53	3,88		P treatment = ns
	4 months	-22,07	3,65		-17,77	3,95		-20,75	4,60		p time = ns
**STI derived EF (%)**	1 month	58,8	9,78	5	45,14	11,07	7	58,70	6,98	10	P interaction = ns
	2 months	54,4	6,23		45,71	11,40		50,60	11,30		P treatment = *
	4 months	52	4,64		37,71	8,04		51,80	8,99		p time = **
**FAC (%)**	1 month	37,83	6,43	6	32,71	9,43	7	40,08	7,90	12	P interaction = ns
	2 months	38,83	7,22		33,71	7,20		33,92	7,84		P treatment = ns
	4 months	41,00	5,59		33,71	5,19		38,83	7,15		p time = ns

Table S1. Conventional and strain measures of echocardiography.

Measurements were performed on PLAX (parasternal long axis) and PSAX (parasternal short axis) views. Conventional parameters were evaluated from bidimensional images (B-Mode) by outlining the endocardium using Vevolab software: HR, heart rate; Area;s, Left ventricular end-systolic area, Area;d, Left ventricular end-systolic area, volume;s, left ventricular end-systolic volume; volume;d, left ventricular end-diastolic volume; SV, stroke volume; EF, left ventricular ejection fraction, FS, longitudinal fraction shortening. Speckle tracking imaging (STI) allowed measuring strain parameters

using Vevostrain software: global long. strain, global longitudinal strain (peak), global circ. strain, global circumferential strain (peak), STI derived EF, speckle tracking imaging-derived ejection fraction; FAC, fractional area change.

Values are presented as the mean ± SD. Statistical analysis was performed using Two-way ANOVA (*GraphPad Prism*). Statistical significance was noted: p=ns for p>0.05, p* for p<0.05, p** for p<0.01, p*** for p<0.001 and p**** for p<0.0001.

Supplementary references

1 Boisguerin, P. *et al.* Systemic delivery of BH4 anti-apoptotic peptide using CPPs prevents cardiac ischemia-reperfusion injuries in vivo. *J Control Release* **156**, 146-153, doi:10.1016/j.jconrel.2011.07.037 (2011).

2 Roubille, F. *et al.* Myocardial expression of a dominant-negative form of Daxx decreases infarct size and attenuates apoptosis in an in vivo mouse model of ischemia/reperfusion injury. *Circulation* **116**, 2709-2717, doi:10.1161/CIRCULATIONAHA.107.694844 (2007).

3 Roubille, F. *et al.* Delayed postconditioning in the mouse heart in vivo. *Circulation* **124**, 1330-1336, doi:10.1161/CIRCULATIONAHA.111.031864 (2011).

4 Vincent, A. *et al.* Down-regulation of the transcription factor ZAC1 upon pre- and postconditioning protects against I/R injury in the mouse myocardium. *Cardiovasc Res* **94**, 351-358, doi:10.1093/cvr/cvr310 (2012).

5 Vincent, A. *et al.* Cardiac mGluR1 metabotropic receptors in cardioprotection. *Cardiovasc Res*, doi:10.1093/cvr/cvx024 (2017).

6 Reardon, S. A mouse's house may ruin experiments. *Nature* **530**, 264, doi:10.1038/nature.2016.19335 (2016).

7 Fattah, C. *et al.* Gene Therapy With Angiotensin-(1-9) Preserves Left Ventricular Systolic Function After Myocardial Infarction. *Journal of the American College of Cardiology* **68**, 2652-2666, doi:10.1016/j.jacc.2016.09.946 (2016).

8 Piot, C. A., Padmanaban, D., Ursell, P. C., Sievers, R. E. & Wolfe, C. L. Ischemic preconditioning decreases apoptosis in rat hearts in vivo. *Circulation* **96**, 1598-1604 (1997).

9 Reagan-Shaw, S., Nihal, M. & Ahmad, N. Dose translation from animal to human studies revisited. *FASEB journal : official publication of the Federation of American Societies for Experimental Biology* **22**, 659-661, doi:10.1096/fj.07-9574LSF (2008).

10 Heurteaux, C. *et al.* Neuroprotective and neuroproliferative activities of NeuroAid (MLC601, MLC901), a Chinese medicine, in vitro and in vivo. *Neuropharmacology* **58**, 987-1001, doi:10.1016/j.neuropharm.2010.01.001 (2010).

11 Chen, C. *et al.* Danqi Piantang Jiaonang (DJ), a traditional Chinese medicine, in poststroke recovery. *Stroke* **40**, 859-863, doi:10.1161/strokeaha.108.531616 (2009).

12 Leitman, M. *et al.* Two-dimensional strain-a novel software for real-time quantitative echocardiographic assessment of myocardial function. *Journal of the American Society of Echocardiography : official publication of the American Society of Echocardiography* **17**, 1021-1029, doi:10.1016/j.echo.2004.06.019 (2004).
